# Oat-Based Vegan Yogurt Alternatives Enriched with Mung Bean and Black Bean Protein Isolates: Nutritional and In Silico Potential Bioactive Peptide Estimation

**DOI:** 10.3390/foods15162825

**Published:** 2026-08-13

**Authors:** Murat Emre Terzioğlu, Elif Ekiz Terzioğlu

**Affiliations:** Department of Food Engineering, Faculty of Agriculture, Atatürk University, Erzurum 25240, Türkiye; elifekiz@atauni.edu.tr

**Keywords:** oat-based vegan yogurt, mung bean, black bean, protein isolates, nutritional composition, peptidomic

## Abstract

Although the development of fermented plant-based milk alternatives has become quite popular, the relatively low nutritional value and functional properties of these products present significant challenges. This situation is driving the search for alternative protein sources to improve the quality of plant-based products, and legume proteins are attracting attention as promising components. Accordingly, this study aimed to enrich oat-based vegan yogurt alternatives with different legume protein isolates and evaluate their effects on product quality. In the present study, physicochemical, microbiological, antioxidant capacity, FAA profile, peptide profile, and FTIR analyses were performed on vegan yogurts produced using oat milk as well as mung bean and black bean protein isolates. It was determined that different plant protein isolates used in vegan yogurt production had a statistically very significant (*n* = 2, *p* < 0.01) effect on dry matter, protein, pH, *L. acidophilus*, antioxidant capacity, and all FAA profiles except asparagine and aspartic acid. The addition of legume protein isolates to vegan yogurts contributed to an increase in dry matter content (11.57–14.14%), protein content (2.92–5.20%), ash content (0.22–0.32%), and pH value (4.49–4.59) compared to the control group. Similar effects were also observed in *S. thermophilus* count (5.79–6.38 log cfu/g), *L. delbrueckii* subsp. *bulgaricus* count (5.72–6.43 log cfu/g), DPPH inhibition (10.29–19.93%), and ABTS inhibition (14.66–28.48%). It was determined that the addition of mung bean protein isolate (MBPI-2 and MBPI-4), compared to black bean protein isolate, contributed more to nutritional properties in vegan oat yogurt samples. Vegan yogurts were found to match more frequently with antioxidant, ACE inhibitor, DPP III, and DPP IV inhibitor activity in potential bioactivity predictions. FTIR analysis revealed that using different legume protein isolates in vegan yogurt production did not result in the formation of new absorption bands, but it did affect the intensity of existing regional bands. In conclusion, the addition of different legume protein isolates to oat yogurt was found to generally contribute positively to the parameters examined and represents a promising approach for individuals who cannot consume cow milk due to health problems, as well as for vegans.

## 1. Introduction

Today, changes in dietary habits and consumer awareness have made plant-based diets popular, increasing demand for innovative, sustainable, and nutritious plant-based products as alternatives to animal-derived foods. In human nutrition, milk and dairy products are among the foods that contain a balanced amount of macro and micronutrients essential for life. On the other hand, developing alternative products is important for people suffering from lactose intolerance, cholesterol, malabsorption, or milk protein allergy [1,2]. Indeed, the global dairy alternatives market was valued at US$32.77 billion in 2024 and is projected to reach US$66.91 billion by 2030, with a compound annual growth rate of 12.7% [3]. Fermentation applications to improve the nutritional profile, sensory and functional properties of plant-based milk alternatives meet the demands of consumers and the food industry with a natural and effective biotechnological approach [4]. Plant-based milks exhibit a weaker nutritional profile compared to cow’s milk. Therefore, offering alternative products made from plant-based milks that are similar to those made from animal milk and rich in nutrients provides a solution to the search for quality alternative products for vegan individuals [1]. With a content of 49–63.5% carbohydrates, 4.5–7.4% protein, 3.2–4.4% dietary fiber, and 0.01% cholesterol, oats are a recognized source among plant-based raw materials used in milk production. However, the fact that oats are rich in beta-glucan, avenantramidine, tocopherols, and tocotrienols makes oat milk even more valuable in terms of nutrients and bioactive components [5,6].

In vegan yogurt production, the use of hydrocolloids such as starch improves texture by reducing syneresis through their water-binding ability, while protein enrichment also increases firmness by reducing syneresis through cross-linking with fat globules. Additionally, the interaction of hydrocolloids with proteins strengthens the network structure and increases viscosity. In this context, the use of starch and protein as texture enhancers plays a critical role in improving mouthfeel and spoonability characteristics in vegan yogurt production [7].

Mung beans (*Vigna radiata* L.), which have attracted particular attention compared to other legumes in recent years, have a production and consumption history of more than 3500 years and are originally from India and Central Asia. The interest in mung beans stems from their 20–32% protein content, and this high protein content confers various techno-functional properties such as emulsifying, gelling and foaming, and oil and water binding capacity [8]. Similarly, black beans (*Phaseolus vulgaris* L.) contain 17.9–31.1% protein, and their amino acid and bioactive peptide content contributes to their functionality. Black beans are notable for their high-quality protein content, similar to meat, milk, and eggs, and are also an important source of anthocyanins, isoflavones, vitamin E, carotenoids, and saponins. Black bean protein isolates are also reported to possess techno-functional properties such as emulsifying, foaming, and solubility, similar to mung bean isolates [9,10].

In light of all this information, improving the relatively limited nutritional profile of plant-based milks makes it important to develop new formulations with enhanced nutritional and functional properties for individuals who cannot consume animal milk or who are vegan. In this context, the current study presents an innovative approach by enriching vegan yogurts produced from oat milk with mung bean and black bean protein isolates. While chickpea or pea proteins are commonly used as plant-based protein sources in vegan yogurt alternatives [11,12], mung bean and black bean proteins were used in this study due to their balanced and high-quality nutritional value, as well as their potential functional properties [13]. Furthermore, to our knowledge, the insufficient utilization of proteins derived from these legumes in plant-based fermented milk alternatives presents a significant research opportunity in the development of functional and high-value-added vegan yogurt products. The aim of this study is to investigate the effects of mung bean protein isolate (MBPI) and black bean protein isolate (BBPI) on the physicochemical and microbiological properties, free amino acid (FAA) profile, antioxidant capacity, and structural changes of oat-based vegan yogurt alternatives. In addition, to assess the suitability of these legume proteins for the development of plant-based yogurt alternatives, the potential bioactivities of the peptides identified by peptidomic characterization were investigated in silico. Thus, the potential of the innovative fermented product developed was examined as a sustainable plant-based yogurt alternative in terms of both its nutritional profile and functional properties.

## 2. Materials and Methods

### 2.1. Material

In this study, oatmeal (Eti Lifalif, Eskişehir, Türkiye), starch (Dr. Oetker, Izmir, Türkiye), mung beans (Duru Bulgur, Karaman, Türkiye), black beans (Yayla, Mersin, Türkiye), and probiotic yogurt culture (Danem, Isparta, Türkiye), which were used as raw materials in the production of vegan yogurt, were sourced from local markets in Erzurum (Türkiye). According to the label information provided, 100 g of mung beans contain 48 g of carbohydrates, 1.4 g of fat, 22.1 g of protein, and 0.30 g of salt, while 100 g of black beans contain 61.6 g of carbohydrates, 2.4 g of fat, 21.1 g of protein, and 0.1 g of salt. According to label information, 100 g of oats contain 55 g of carbohydrates, 7.2 g of fat, 15 g of protein, and 0.01 g of salt.

#### Reagents

2,2-Diphenyl-1-picrylhydrazyl, 2,2′-Azino-bis(3-ethylbenzothiazoline-6-sulfonic acid), methanol, potassium persulfate, bile salt, and sodium hydroxide were obtained from Sigma-Aldrich (Hamburg, Germany); sulfuric acid and formic acid from Isolab (Eschau, Germany); hydrochloric acid and Kjeldahl tablets from Supelco (Bellefonte, PA, USA); boric acid from Merck (Darmstadt, Germany); and M17 agar and MRS agar from Oxoid (Basingstoke, UK).

### 2.2. Methods

#### 2.2.1. Oat Milk Production

Oat milk production was carried out according to the method described by Łopusiewicz, with some modifications [14]. In this process, oatmeal was mixed with pure water in a 1:10 ratio and left to stand at room temperature for 2 h. Next, the oatmeal mixture was homogenized in a food processor (Musullu MSL-2050 BL-MAX 3000 W, Musullu Elektronik, Antakya, Türkiye) for 3 min (medium speed, approximately 15,000 rpm), and the mixture was filtered using two layers of cheesecloth.

#### 2.2.2. Production of Protein Isolates

The alkaline dissolution–isoelectric precipitation method was used in the production of the protein isolates. After homogenizing the beans with pure water at a ratio of 1:10, the pH of the mixture was adjusted to 9.5 with 1 N NaOH and stirred for 1 h. After centrifuging the mixture at 10,000 *g* for 20 min, the supernatant was collected and the pH was adjusted to 4.5 with 1 N HCl. After centrifugation, the collected pellets were lyophilized and used in the production of vegan yogurt [15]. In addition, the protein content of the protein isolates was determined using the Kjeldahl method [16]. Accordingly, the protein content of mung bean protein isolates was determined to be 90.43%, while the protein content of black bean protein isolates was 80.16%.

#### 2.2.3. Vegan Yogurt Production

**Figure 1:**
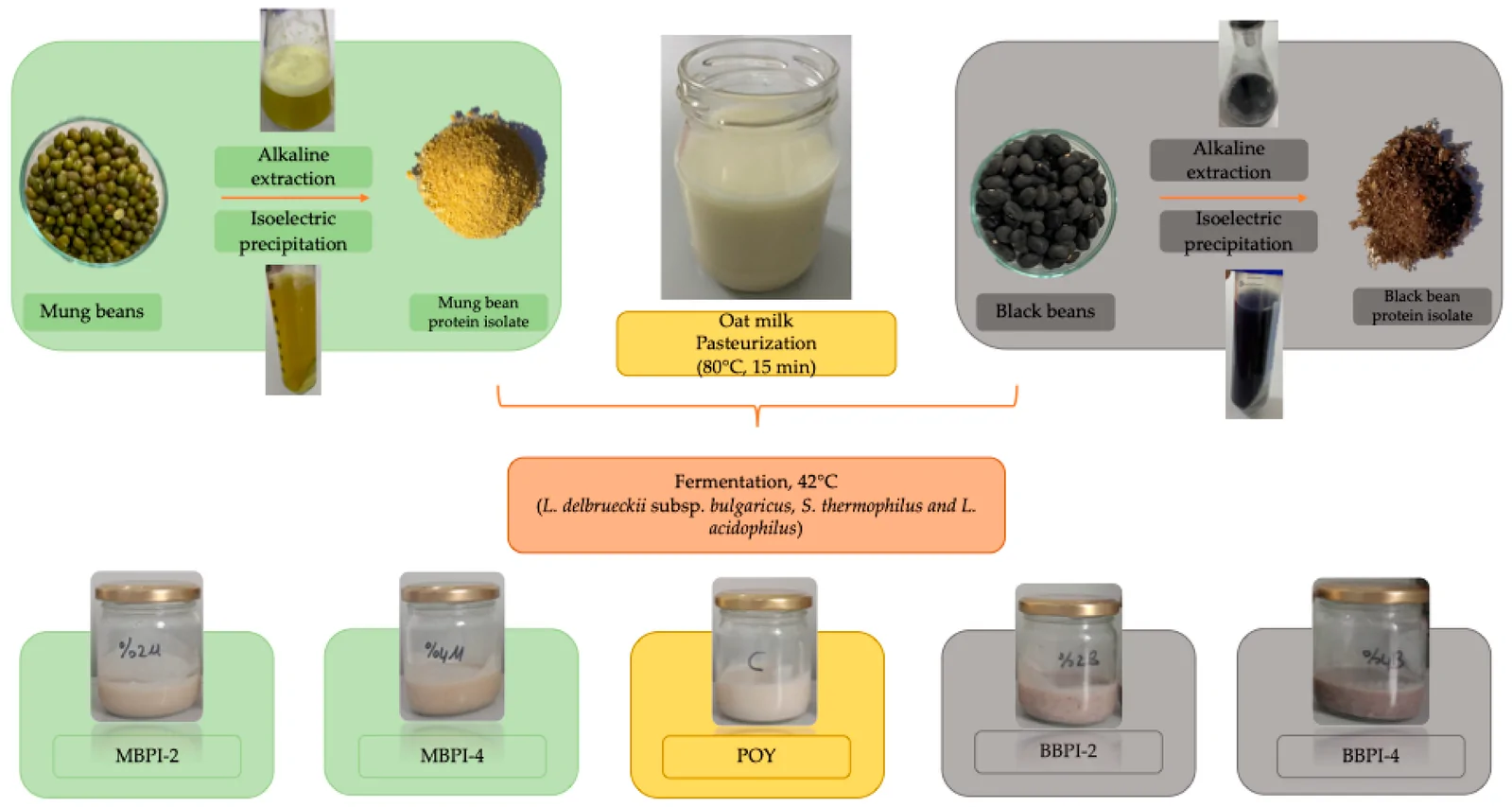
Production of oat-based vegan yogurts enriched with various legume protein isolates.

To produce the vegan yogurts, the oat milk was first divided into five groups, with the control group receiving only 0.05% starch. The other groups received 0.05% starch and different concentrations (2% and 4%) of mung bean and black bean isolates. After the oat milk was formulated, the mixtures were heat-treated at 80 °C for 15 min to improve starch gelatinization, protein interaction, viscosity, and microbial safety. The mixtures were then cooled to 42 °C, inoculated with commercial probiotic yogurt culture (*L. delbrueckii* subsp. *bulgaricus*, *S. thermophilus* and *Lactobacillus acidophilus*, 1 × 10^6^ cfu/g) at a rate of 0.5 g per liter of mixture, and incubated until the pH reached 4.6. After incubation was completed, vegan yogurt samples were stored at 4 °C for 24 h and used in analyses [17]. All formulations contained the same amount of oat milk, starter culture, and starch, with only the type and concentration of protein isolate differing in the formulations of the oat yogurt samples (Table 1). The production process of oat-based vegan yogurts enriched with various legume protein isolates is shown in Figure 1.

#### 2.2.4. Physicochemical Analyses

Dry matter, protein, ash and pH analyses in vegan yogurts were performed using the methods given by Terzioğlu and Bakirci [18]. In dry matter analysis, approximately 5 g of sample was kept at 105 °C until a constant weight was reached, while in ash analysis, the organic material was burned (550 °C) and the inorganic material was determined. Protein content was determined using the Kjeldahl method. pH measurements were performed using a calibrated pH meter by immersion.

#### 2.2.5. Microbiological Analyses

M17 Agar (Oxoid Ltd., Hampshire, UK) was used to determine the *S. thermophilus* counts in vegan yogurts (incubation duration and temperature: 42 °C for 24 h). MRS Agar (Oxoid Ltd.) was used to determine the *L. delbrueckii* subsp. *bulgaricus* counts (incubation duration and temperature: 42 °C for 48 h). For determining the *L. acidophilus* counts (incubation duration and temperature: 37 °C for 48 h), MRS Agar (Oxoid Ltd.) + 1.5 g/L bile (Bile salts, Sigma-Aldrich) was used [18].

#### 2.2.6. Antioxidant Capacity

In the antioxidant capacity analysis, methanol was used in the preparation of the extracts; 3 g of vegan yogurt sample was diluted with methanol to 30 mL and centrifuged at 6000 rpm (for 15 min). For the analysis of ABTS radical scavenging activity, 7 mM ABTS solution prepared using 2.45 nM K_2_S_2_O_8_ was added to 100 µL of extract and incubated in the dark for 6 min, and absorbance measurements were performed at a wavelength of 734 nm. For DPPH free radical scavenging activity, 100 µL of extract was taken and DPPH solution was added to bring the total volume to 3 mL. After the mixture was left in the dark for 30 min, measurements were taken at a wavelength of 517 nm [19].

#### 2.2.7. FAA Profile

To determine the free amino acid profile of vegan yogurts, 100 µL of yogurt sample was mixed with 650 µL of solvent and 50 µL of internal standard, and then centrifuged. The obtained extracts were then analyzed using a Liquid Chromatography Tandem Mass Spectrometry System (LC-MS/MS, Agilent 6460 Triple Quadropol, Santa Clara, CA, USA). In LC-MS/MS, gas temperature was set as 150 °C, gas flow as 11 L/min, the nebulizer at 40 psi, the sheath gas heater at 375 °C, sheath gas flow as 11 L/min, capillary voltage as 2000 V positive and 0 V negative, injection volume as 1 µL and ion mode as positive. Limit of Detection (LOD) and Limit of Quantitation (LOQ) values for the examined amino acids are presented in Table 2 [20].

#### 2.2.8. Peptide Profile and In Silico Potential Bioactive Peptide Estimation

For peptide extraction from vegan yogurts, C18 SPE cartridges conditioned with 0.1% formic acid and methanol were used. The peptides were then eluted using formic acid and acetonitrile. Liquid Chromatography–Quadrupole Mass Spectrometry (Accurate-Mass Quadrupole Time of Flight–Q-TOF LC/MS) was used to separate the peptides in the extracts [21,22]. The in silico potential bioactivity estimates of the obtained peptides were determined using sequence matching of bioactive peptides in the BIOPEP database. Potential biological activity estimations were performed using the BIOPEP database, categorized as “Bioactive peptides—Analysis—Potential Biological Activity Profiles—For Your Sequences”. Since the resulting peptide sequences were short-chain, no restrictions were applied during the database search. The BIOPEP analysis module used in this study does not offer various user-configurable lookup parameters such as trust module, identity threshold, confidence level, or cutoff point. In this context, analyses in the database were performed using the default settings of the database mentioned above, without applying any additional user-defined criteria. Matches were evaluated based on the sequences in the identified peptides.

#### 2.2.9. FTIR Analysis

FTIR measurements were performed to examine structural changes at the molecular level in vegan yogurt. FTIR measurements were performed in the 400–4000 cm^−1^ range, with a resolution of 4 cm^−1^, and an average of 20 scans were taken. Analyses were performed in ATR mode, and background spectra were taken before each measurement to minimize water vapor and CO_2_ interference. During the measurement, full contact was ensured between the sample and the ATR crystal, and the crystal surface was cleaned between measurements [23].

#### 2.2.10. Statistical Analysis

This study was conducted using a completely randomized experimental design with two replications, by adding different concentrations (2% and 4%) of mung bean and black bean protein isolate to vegan yogurts produced from oat milk. The results were evaluated using the SPSS 25.0 software package and expressed as mean ± standard deviation. Duncan’s multiple comparison test was used to determine significant differences between samples, with significance levels of *p* < 0.05 for significance and *p* < 0.01 for high significance.

## 3. Results and Discussion

### 3.1. Physicochemical Analyses

The physicochemical analysis results of vegan yogurt samples enriched with protein isolates produced using mung beans and black beans are presented in Table 3. The use of plant protein isolates in vegan yogurt production was found to have a statistically significant (*p* < 0.05) effect on ash content and a very significant (*p* < 0.01) effect on dry matter, protein, and pH values. In all oat yogurt samples, dry matter content ranged from 10.03% to 14.14%, protein content from 2.01% to 5.20%, ash content from 0.11% to 0.32%, and pH values from 4.40 to 4.59. Among the oat yogurt samples, the lowest dry matter, protein, ash, and pH values were found in the POY samples. It was determined that the addition of black bean protein isolate to oat yogurt samples had a more positive effect on dry matter and ash values compared to mung bean protein isolate. On the other hand, in terms of protein and pH values of oat yogurt samples, the mung bean protein isolate stood out more. Since mung bean protein isolates (90.43%) have a higher protein content compared to black bean protein isolates (80.16%), it is actually an expected result that mung bean protein isolate would provide a more significant increase in protein content when added to oat yogurt. In fact, the highest dry matter and ash values were determined in the BBPI-4samples, and the highest protein and pH values were determined in the MBPI-4samples.

Kudre et al. [13] reported that mung bean protein isolates had a protein content of approximately 87.83%, an ash content of approximately 4.09%, and a moisture content of approximately 3.61%, while black bean isolates had a protein content of approximately 88.21%, an ash content of approximately 3.98%, and a moisture content of approximately 3.52%. The researchers reported that protein content exceeding 70% in the protein isolates they produced met the criteria for legume protein isolates. In addition, they reported that differences in the protein content of protein isolates may be influenced by differences in the protein content of plant sources isolated under alkaline conditions, as well as by the different ways in which other components precipitate together at pH 4.5 during protein recovery. They stated that the differences in ash content of protein isolates may be due to the strong alkalis and acids used for pH adjustment during the protein extraction process. Brishti et al. [24] investigated the effects of freezing, spray drying, and oven drying after protein extraction on the protein content of mung bean protein isolate. The researchers reported that the highest protein content was obtained through freeze-drying, while spray and oven drying processes caused protein swelling due to protein denaturation, leading to the formation of compounds that were no longer detectable as nitrogen-based components in the Kjeldahl analysis. Bulut et al. [25] reported that in yogurt samples produced by adding 0.5% and 1% chickpea and pea proteins, the pH values varied between 4.44 and 4.58 on the first day of storage, and that the buffering capacity caused by the proteins was effective in the fluctuation of pH values.

### 3.2. Microbiological Analyses

It has been stated that *S. thermophilus*, *L. delbrueckii* subsp. *bulgaricus*, *L. acidophilus*, and *Lactiplantibacillus plantarum* are generally used in plant-based yogurt production, and that the survival rate of streptococci and lactobacilli in plant-based products is directly affected by the specific starter cultures used and the product characteristics [11,26,27,28,29]. Therefore, *S. thermophilus*, *L. delbrueckii* subsp. *bulgaricus*, and *L. acidophilus* bacteria were used in the present study. The microbiological analysis results of vegan yogurt samples enriched with protein isolates produced using mung beans and black beans are presented in Table 4. The use of plant protein isolates in vegan yogurt production was found to have a statistically significant (*p* < 0.05) effect on the numbers of *S. thermophilus* and *L. delbrueckii* subsp. *bulgaricus*, and to lead to very significant (*p* < 0.01) differences in the number of *L. acidophilus*. In all oat yogurt samples, the counts of *S. thermophilus*, *L. delbrueckii* subsp. *bulgaricus*, and *L. acidophilus* were found to range from 5.61 to 6.38 log cfu/g, 5.53 to 6.43 log cfu/g, and 5.58 to 6.11 log cfu/g, respectively. On the other hand, live *L. acidophilus* counts in MBPI-2 and BBPI-2 samples remained above the minimum level of 6 log cfu/g, indicating that these formulations have the potential to serve as carriers for live probiotic bacteria. Furthermore, it was shown that the addition of 2% plant protein isolate led to a greater increase in the numbers of *S. thermophilus*, *L. delbrueckii* subsp. *bulgaricus*, and *L. acidophilus*, and further supported microbial growth. On the other hand, it was found that the addition of 4% plant protein isolate reduced the growth of *S. thermophilus*, *L. delbrueckii* subsp. *bulgaricus*, and *L. acidophilus*. Here, it was hypothesized that differences in the amino acid profiles of vegan yogurts enriched with different legume protein isolates might influence changes in microbial viability. Indeed, it has been reported by Savijoki et al. [30] and Huang et al. [31] that protein-derived amino acids are the primary nitrogen source in the growth of lactic acid bacteria and play a role in various physiological processes such as peptide biosynthesis or cellular metabolism. Furthermore, phenolic compounds such as anthocyanins and flavonoids are known to possess antimicrobial activities through pathways such as interference with microbial metabolism or membrane disruption. In this context, it was considered that these antimicrobial compounds may have a potential effect on the reductions observed in the number of microorganisms with the addition of higher percentages of plant protein isolates [32].

In the present study, it was determined that the numbers of *S. thermophilus* and *L. delbrueckii* subsp. *bulgaricus* showed a similar trend, decreasing and increasing together in all oat yogurt samples. This situation is thought to result from a symbiotic relationship between the two bacteria. *L. delbrueckii* subsp. *bulgaricus* supports the growth of *S. thermophilus* through the peptides and amino acids it produces with its high proteolytic activity, while *S. thermophilus* creates a suitable environment for the growth of *L. delbrueckii* subsp. *bulgaricus* by producing formic acid, pyruvate, and CO_2_ [33]. Accordingly, it can be interpreted that when the protein content exceeds a certain level, *L. delbrueckii* subsp. *bulgaricus*, which has high proteolytic activity, provides a greater amount of peptides and amino acids to the environment, resulting in the substrate exceeding the optimum level for *S. thermophilus*, which has limited proteolytic activity, potentially disrupting the symbiotic relationship. Therefore, based on all this data, it has been shown that enriching yogurt with the correct amount of protein is important for bacterial growth. On the other hand, in all oat yogurt samples except BBPI-2, the number of *S. thermophilus* was found to be higher than the number of *L. delbrueckii* subsp. *bulgaricus*. This situation is explained by the fact that *S. thermophilus* is more competitive in substrate utilization compared to *L. delbrueckii* subsp. *bulgaricus* [34]. Similarly, in all oat yogurt samples, it was observed that the numbers of *L. delbrueckii* subsp. *bulgaricus* and *L. acidophilus* varied in parallel. This is thought to be due to the synergistic effect between *L. delbrueckii* subsp. *bulgaricus* and *L. acidophilus*, as well as the high proteolytic activity of *L. delbrueckii* subsp. *bulgaricus*, which can provide even more amino acids necessary for its survival [35]. The slightly lower numbers of *S. thermophilus*, *L. delbrueckii* subsp. *bulgaricus*, and *L. acidophilus* in yogurts produced from oat milk, compared to some studies in the literature on yogurts produced from different animal milks [18,36,37], are thought to be due to the composition and structure of oat milk. In fact, oat-based products have lower lactic acid concentrations and lower buffering capacity at the same pH levels compared to cow milk, which can limit the growth of lactic acid bacteria during the fermentation process [38].

### 3.3. Antioxidant Capacity

The antioxidant capacity analysis (inhibition % and Trolox equivalent) results of vegan yogurt samples enriched with protein isolates produced using mung beans and black beans are presented in Table 5. The use of plant protein isolates in vegan yogurt production was found to have a statistically very significant (*p* < 0.01) effect on DPPH radical scavenging activity and ABTS radical scavenging activity values. The addition of mung bean and black bean protein isolates was found to increase the antioxidant capacity of oat yogurt samples. In fact, the lowest values for both DPPH radical scavenging activity (inhibition % and Trolox equivalent) and ABTS radical scavenging activity (inhibition % and Trolox equivalent) were found in POY samples. However, although the addition of MBPI and BBPI was generally observed to increase antioxidant capacity compared to the control group, the extent of improvement was primarily related to the protein source and concentration. On the other hand, it is thought that these changes do not follow a strictly dose-dependent pattern and that antioxidant activity is not solely related to protein content but rather to the amino acid profile or peptide composition of the protein isolate.

The radical scavenging activities of antioxidants in foods arise as a result of the antioxidants’ ability to participate in single electron transfer reactions, and an increase in peptidic amino acid residues capable of transferring electrons to free radicals contributes to an increase in antioxidant activity properties [39,40]. It has been reported that proline, histidine, methionine, cysteine, and aromatic amino acids are effective in the antioxidant capacity of food peptides, and that the antioxidant property of peptides is also increased by adding hydrophobic amino acids, leucine and proline, to the N-terminus of His-His dipeptide [41,42]. It has also been reported that phenylalanine can scavenge OH radicals to form stable para-, meta-, or ortho-substituted hydroxylated derivatives, and that peptide tryptophan, tyrosine, and phenylalanine residues can contribute to the chelation of pro-oxidant metal ions [43]. Additionally, it has been stated that the structural properties of amino acids, such as the proton-donating ability of histidine from its imidazole group and the hydrogen donor properties of tyrosine due to its phenolic groups, also contribute positively to antioxidant activity [44]. Kou et al. [45] and Torres-Fuentes et al. [46] stated that plant protein hydrolysates have many antioxidant effects, such as scavenging free radicals, delaying reactive oxygen species, preventing peroxide radicals of lipid oxidation, and/or chelating pro-oxidation transition metals. Butseekhot et al. [47] investigated the antioxidant activities of black bean and red kidney bean protein concentrates obtained by alkaline extraction. The researchers reported that black bean protein concentrates had higher scavenging activities for ABTS^•+^, DPPH^•^ and Fe^3+^ radicals. It has been emphasized that oat-based products such as oat milk and oat yogurt also have effective antioxidant activity due to the phenolic compounds, vitamins, and bioactive phytochemicals they contain [48]. Additionally, Alemayehu et al. [49] stated that oats have a very rich composition in terms of avenanthramides, which have 10–30 times higher antioxidant activity than the polyphenolic compounds of other cereals, such as ferulic acid, vanillin, vanillic acid, syringic acid, gentisic acid, protocatechic acid and p-hydroxybenzoic acid. On the other hand, Uruc et al. [50] and Terzioğlu et al. [51] showed that the antioxidant capacity of fermented milk products is increased due to the formation of amino acids and low molecular weight peptides as a result of proteolysis occurring during fermentation.

### 3.4. FAA Profile

The importance of amino acids actually stems from their roles in metabolic activities in the body. For example, proline plays an important role in wound healing, skin health, and collagen production; arginine in the circulatory system; tyrosine in the synthesis of neurotransmitters; cysteine in glutathione synthesis; alanine in glucose metabolism and energy production; glutamic acid in brain health; glycine in the regulation of inflammation and the immune system; serine in the immune response and muscle formation; methionine in methylation processes; and tryptophan in serotonin synthesis [52,53]. The FAA results of vegan yogurt samples enriched with protein isolates produced using mung beans and black beans are presented in Table 6. The use of plant protein isolates in vegan yogurt production was found to have a statistically very significant (*p* < 0.01) effect on all free amino acid values examined, except for asparagine and aspartic acid. In all yogurt samples, the addition of plant protein isolate was found to increase the levels of arginine, phenylalanine, histidine, leucine, methionine, threonine, tryptophan, valine, alanine, asparagine, aspartic acid, glycine, glutamine, proline, and serine amino acids. On the other hand, it was determined that the addition of mung bean protein isolate to oat yogurt samples (in proportion to the addition rate) increased the values of arginine, phenylalanine, isoleucine, lysine, leucine, methionine, threonine, tryptophan, valine, alanine, asparagine, glycine, glutamine, proline, serine, and tyrosine compared to the addition of black bean protein isolate. In fact, excluding aspartic acid and cystine, the highest FAA values were detected in the MBPI-4 sample. Except for isoleucine, lysine, glutamic acid, cystine, and tyrosine, the lowest FAA values were determined in the POY sample.

The significant increase in FAA content in oat yogurts enriched with different legume protein isolates, compared to the control group, is thought to be primarily due to the high protein content of mung bean protein isolate (90.43%) and black bean protein isolate (80.16%). Although oats are rich in sulfur-containing amino acids such as methionine and cysteine, they are quite poor in some amino acids such as lysine [54]. On the other hand, mung bean proteins [55] and black bean proteins [56] have a rich amino acid profile. In this context, the combined use of cereal and legume proteins in vegan yogurt production had a complementary effect on FAA, significantly improving the nutritional value of vegan yogurts. However, the fact that the FAA composition was generally higher in the groups including mung bean protein isolate compared to the groups including black bean protein isolate in this study can be attributed to the differences in the protein structures, amino acid compositions and ratios, and digestibility of legumes. Indeed, Kudre et al. [13] confirmed that there are significant differences in the amino acid compositions of mung bean and black bean protein isolates. The proteolytic activity of lactic acid bacteria is another influential factor on changes in the amino acid composition of fermented products. Fermentation is characterized by a significant improvement in protein quality, enabling an increase in the relative protein content of both crude and soluble proteins through metabolic processes such as selective consumption of carbohydrates and lipids and partial microbial protein synthesis. Moreover, microbial proteases catalyze the conversion of insoluble proteins into smaller peptides and amino acids, contributing to the exposure of hydrophobic and charged groups, which ultimately contribute to solubility, digestibility, and functional properties [57,58].

On the other hand, the amino acid profile of dairy products essentially begins with the denaturation of proteins during the heat treatment of raw milk. Following this, during fermentation, *S. thermophilus* specifically metabolizes branched-chain amino acids such as leucine, valine, and isoleucine, as well as sulfur-containing amino acids such as cystine and methionine, in addition to glutamic acid, tryptophan, histidine, arginine, and tyrosine [59]. However, Sun et al. [60] reported that the total amino acid content in oats before fermentation was 132.34 mg/g, and that this amount decreased to 69.80 mg/g and 106.04 mg/g in fermentation processes using different cultures. It has been reported that this decrease is due to amino acids being the primary nitrogen source for the growth of microorganisms during fermentation, and the conversion of amino acids into various aroma compounds such as aldehydes, carboxylic acids, and alcohols. However, since amino acid levels are given in ng/mL in this study, and due to differences in units or analytical methods, direct numerical comparisons among studies should be interpreted with caution. On the other hand, the contribution of microorganisms to the production of smaller peptides and free amino acids from proteins through their proteolytic activities in fermentation should not be overlooked. In this context, although amino acids are metabolized by microorganisms, this study suggested that supplementing the vegan yogurt process with protein isolate contributed to a significant increase in amino acid content beyond microbial consumption. Dubois-Laurin et al. [61] reported that in mung bean protein isolates obtained by isoelectric precipitation, the essential amino acids phenylalanine, isoleucine, leucine, and valine, and the non-essential amino acids arginine, proline, and serine stood out. The researchers have reported that the dominance of certain amino acids in protein isolates is influenced by factors such as the decrease in the net charge of proteins at the isoelectric point, the tendency of certain protein fractions to aggregate and precipitate, and the retention and removal of soluble components in the supernatant.

### 3.5. Peptide Profiling and In Silico Potential Bioactivity Estimation

All peptide profile results for vegan yogurts produced from oat milk and enriched with different legume-derived protein isolates are given in Supplementary Table S1. However, the total number of peptides detected in all samples and the number of peptides associated with antioxidant activity are presented in Table 7. Peptide profiling analysis performed using Q-TOF LC/MS revealed 62, 67, 81, 33, and 57 peptides consisting of two to four amino acids in POY, MBPI-2, MBPI-4, BBPI-2, and BBPI-4 samples, respectively. Accordingly, this suggests that proteolytic degradation, which occurs through fermentation in different legume protein isolates, plays a role in the differences in the peptide numbers of the samples. The short-chain nature (two to four amino acids) of the peptides identified here makes reliable identification of protein origins difficult. Accordingly, the determined peptide sequences were evaluated in terms of their potential bioactivities in the BIOPEP-UWM database (https://biochemia.uwm.edu.pl/biopep-uwm/ (accessed on 10 July 2026)). The peptides identified in the control group and in vegan yogurts enriched with different legume isolates were mostly observed to match ACE inhibitor, DPP-IV, antioxidant, and DPP-III inhibitor activity motifs. However, since the identified peptides match numerous potential bioactivities in the database (Supplementary Table S1), only peptides associated with antioxidant activity are presented here to improve readability and interpretability. Furthermore, it is important to emphasize that the presented results reflect predictions based on sequence matching with previously identified bioactive motifs in the BIOPEP database, rather than experimentally validated biological activities. In this context, it is also important to note that the results obtained from the BIOPEP database are not evidence confirming experimentally determined biological activities, but rather should be considered as “potential mechanistic evidence” supporting the possible molecular basis of these activities. Similar challenges and limitations in the identification and bioactivity estimation of short peptide sequences determined in foods have also been reported by Agyei et al. [62] and Minkiewicz et al. [63]. While GPRR, GSKC, ILDV, KAIT, PSVI, VVW, and WGL peptides were found to be common in all vegan yogurts, GPRR, KAIT, VVW, and WGL sequences were observed to exhibit potential antioxidant properties (Supplementary Table S1). It is thought that these peptides, identified in all samples, may be formed during fermentation as a result of the hydrolysis of globulins, the storage proteins of oats, and other oat proteins, due to the proteolytic activity of lactic acid bacteria [64,65]. Indeed, Rafique et al. [66] reported that oat protein and peptides exhibit many therapeutic properties such as antioxidant, antidiabetic, antihypoxic, antithrombotic, antihypertensive, hypocholesterolemic and immunomodulatory. Furthermore, Koutis et al. [67] confirmed that there are significant differences between unfermented and fermented oat milk in terms of biological activities such as antioxidant activity, anti-inflammatory and antiplatelet activity. On the other hand, the fact that potential antioxidant peptides IRF, IRI, LIWY, PVWW, RHYW, and WTY were identified only in groups with added mung beans, and the DAYC peptide only in groups with added black beans, indicates that different legume protein isolates affect the peptide profile formed by fermentation. Furthermore, this finding shows that legume species were hydrolyzed differently by lactic acid bacteria depending on their different protein structures, and this is effective in releasing various peptides [68,69]. Supplementary Table S1 shows that the potential antioxidant peptides presented generally contain amino acids associated with antioxidant activity, such as tryptophan (W), phenylalanine (F), valine (V), tyrosine (Y), leucine (L), histidine (H), and cysteine (C). Aromatic amino acids such as Y and W, and sulfur-containing amino acids such as C, are reported to act as hydrogen or electron donors in the scavenging of free radicals through the indole and phenolic side chains in their structures [9]. Furthermore, it has been reported that the amino acids at the N and C terminals of the sequence are important for antioxidant activity, with the presence of L or A at the N terminal increasing the concentration of the peptide in water/lipid environments, thus providing high antioxidant activity [70]. Previous studies have reported that molecular weight, amino acid type, and sequence in antioxidant peptides are closely related to antioxidant capacity. In free radical scavenging, hydrophobic amino acids (Gly, Val, Ala, Phe, Leu, Pro, and Ile) contribute to the activity by increasing their interaction with free radicals. However, it was reported that histidine and arginine play a role via the imidazole group in their side chains, while proline plays a role via the pyrrolidine ring [70,71,72].

In addition, it was thought that the short peptide chains obtained in the study, and therefore their low molecular weight, as well as the type of amino acids in the peptides, also play a role in antioxidant activity. Indeed, the effect of low molecular weight on antioxidant activity has also been reported by Kusumah et al. [73] and Wang et al. [74]. Additionally, Kusumah et al. [73] reported that the presence of hydrophobic residues affects antioxidant activity in mung bean protein hydrolysates and that the 30 kDa mung bean albumin sequence contains biologically active fragments in the BIOPEP database, namely HL, AY, EL, IR, LK, KP, VY, and FC. In addition, Xia et al. [75] confirmed that mung bean peptides with the sequences Trp-Gly-Asn, Ala-Trp, Arg-Gly-Trp-Tyr-Glu and Gly-Val-Pro-Phe-Trp have high antioxidant capacity. For black beans, Flores-Medellín et al. [76] reported changes in the number and sequences of peptides identified after 48 and 96 h of fermentation, and that amino acids such as T, W, G, S, R, A, and P frequently appeared in the sequence. Furthermore, Mojica and de Mejía [77] characterized the antidiabetic (DPP-IV, α-amylase and α-glucosidase) activity of EGLELLLLLLAG, AKSPLF, FEELN, WEVM peptides obtained from black bean protein hydrolysates through hydrogen bonds, polar and hydrophobic interactions. In the current study, the highest DPPH and ABTS free radical scavenging activity values were observed in the group supplemented with 4% mung bean isolate. However, a greater number of potential antioxidant peptides were identified in the groups supplemented with mung bean isolate compared to the others, suggesting that these peptides may have positive effects on antioxidant activity. This indicates that the antioxidant peptide profile, assessed in silico, is generally consistent with the experimental results for DPPH and ABTS free radical scavenging activity. On the other hand, although the number of potential antioxidant peptides was the same in the group with 2% and 4% mung bean isolate added, the inhibition values were different. Here, however, the relationship is not entirely linear, revealing that antioxidant activity was not solely affected by the number of identified peptides, but also by the type and sequence of amino acids in the peptides and their radical scavenging capabilities [78,79,80]. Furthermore, it should not be overlooked that antioxidant activity was not only influenced by antioxidant peptides but also by other antioxidant compounds in the matrix [67]. In conclusion, it was assessed that the antioxidant capacity determined in vegan yogurts derived from oat milk cannot be explained solely by the number of potential antioxidant peptides, but is shaped by the combined effect of the number and sequence of amino acids in the peptides, the molecular weight of the peptide, and other antioxidant compounds present in the matrix.

### 3.6. FTIR Analysis

**Figure 2:**
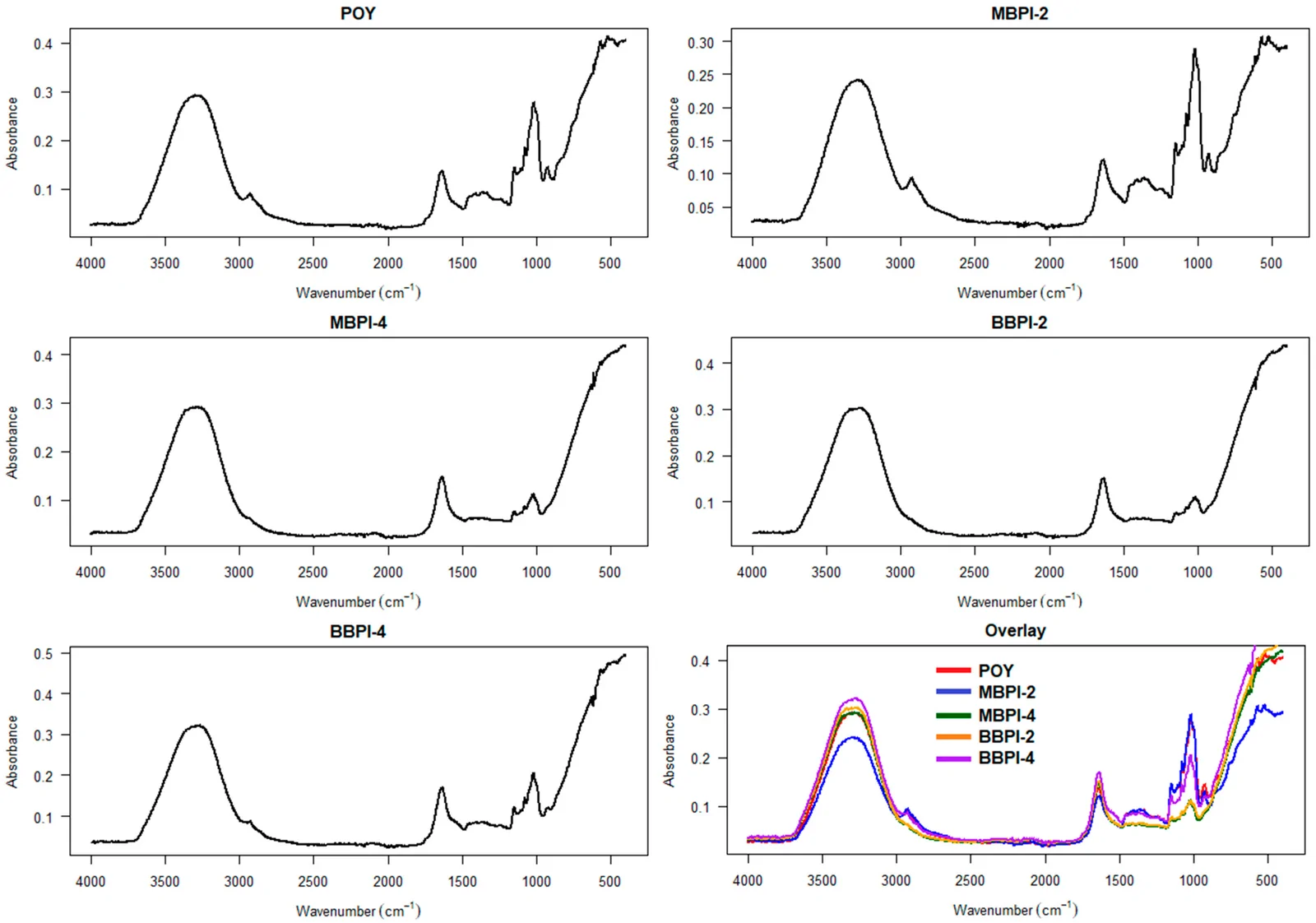
FTIR spectra of oat-based vegan yogurt.

The FTIR spectra of oat-based vegan yogurt are given in Figure 2. When FTIR spectra were evaluated, it was observed that enriching oat-based vegan yogurts with different ratios of mung bean and black bean protein isolates did not contribute to the formation of new absorption bands. This indicates that the addition of protein isolates did not introduce any new functional groups into the fermented oat matrix. In the samples, no new functional groups were added, but with the addition of 2% and 4% of different legume-derived protein isolates, it was observed that the relative intensities in the bands related to characteristic protein and carbohydrate groups changed, and changes also occurred in fermentation and molecular interactions. The broad absorption band between 3200 and 3400 cm^−1^ characterizes O–H and N–H stretching vibrations [81], and this band exhibited a higher relative intensity in vegan yogurts enriched with different legume protein isolates compared to the control group. Essentially, this region (3200 to 3400 cm^−1^) was attributed to stronger hydrogen bonds between proteins, water, and polysaccharides [82]. As expected, the slight increase in band strength observed with the addition of protein isolate suggests an increase in intermolecular hydrogen bond interactions due to the incorporation of bean proteins into the oat gel matrix. The spectrum of approximately 1650 cm^−1^ characterizes the Amide I band, which is associated with C=O stretching vibrations of peptide bonds [83], and this region was more prominent in samples enriched with protein isolate. This suggests that possible rearrangements in the secondary structure of proteins and strengthening of protein–protein interactions were at play. Dinali et al. [84] reported that in the Amid I region, the 1615–1640 and 1689–1698 cm^−1^ peaks belong to β-sheets, the 1650–1659 cm^−1^ peaks belong to α-helices, the 1641–1649 cm^−1^ peaks belong to random helices, and the 1660–1688 cm^−1^ peaks belong to β-spins. Furthermore, they associated the significant increases in β-sheet and α-helix content, despite the decrease in random helices in legume isolates, with an increase in hydrogen bond interactions between protein molecules. In oat-based vegan yogurt production, it is known that lowering the pH reduces electrostatic repulsion between proteins, contributing to aggregation and gel formation. In addition, the proteolytic activity of microorganisms enables the production of peptides that affect the protein network. This result was consistent with previous studies on the structural rearrangement of proteins caused by fermentation. Indeed, Yang et al. [85] reported that during the fermentation of mung proteins, protein molecules are opened and many hydrophobic groups are released, forming hydrophobic aggregates. The Amide II band (~1540 cm^−1^), corresponding to N–H bending and C–N stretching vibrations, showed an increased intensity in vegan yogurts enriched with different legume protein isolates. This suggests that mung bean and black bean protein isolates are effectively integrated into the protein network of the oat yogurt matrix. The simultaneous increase observed in the Amide I and Amide II bands indicates both the effect of the protein isolate enrichment process and the rearrangement of molecules through fermentation. Paz et al. [86] also stated that the most important spectral changes resulting from the breakdown of proteins by the proteolytic activity of microorganisms in yogurt production occur in the Amide I and Amide II regions. Indeed, in this study, the changes observed in the Amide I and Amide II regions may reflect structural reorganization associated with proteolytic activity and protein isolate addition during fermentation. Additionally, it should be emphasized that these interpretations are based on FTIR spectral features and are considered indicative only, not definitive evidence regarding secondary structural changes in proteins. On the other hand, Altan and Yağci [87] reported that during the fermentation of fava bean protein, a decrease in the intensity of the Amide I band was observed as a result of changes in the secondary structure of the proteins, and there was no significant change in the α-helix and β-spin contents, while an increase in random helix was observed. They also reported that protein aggregates and amino acid side chains were effective in the peaks at 1613 cm^−1^ and 1694 cm^−1^. Furthermore, vegan yogurts showed significant differences in the 1200–900 cm^−1^ fingerprint region, which characterizes the C–O, C–O–C, and C–C stretching vibrations of carbohydrates and polysaccharides [88]. In these regional spectra, β-glucan, starch-derived polysaccharides, and soluble dietary fiber, primarily derived from oats, are the key components, while residual carbohydrate fractions that may be present in the isolates can also have an effect. Oats are known for their composition rich in β-glucan and starch [89].

In oat-based vegan yogurt production, acidification during fermentation alters the net charge distribution of proteins, facilitating not only protein–protein interactions but also the formation of protein–polysaccharide complexes (via hydrogen bonds and electrostatic forces). It was also emphasized that protein–polysaccharide interactions are one of the fundamental molecular mechanisms framing the mechanical properties, network stability, and water-holding capacity of food gels [90,91]. In this context, it was thought that the changes in O–H/N–H stretching vibrations in the 3200–3400 cm^−1^ region and the density changes in the 1200–900 cm^−1^ carbohydrate fingerprint regions in the FTIR spectra of vegan yogurt samples are influenced not only by the increase in protein content but also by the reorganization of molecular interactions between these biopolymers. Furthermore, spectral variations in this region can be explained by changes in polysaccharide organization resulting from the metabolism of fermentable carbohydrates. Spectral changes between different legume protein isolates are thought to be influenced by the differences in polysaccharide content and protein structure between mung beans and black beans. Similarly, Greulich et al. [92] associated spectral changes in the carbohydrate fingerprint region with the use of carbohydrates by microorganisms for their growth during fermentation. In conclusion, the use of protein isolate in vegan yogurt production has been shown to increase the density of protein-related absorption bands and their incorporation into the oat matrix depending on the ratio. On the other hand, the combined dynamics of fermentation and legume proteins on molecular changes in vegan yogurt production should be emphasized. Furthermore, the influence of interactions between oat-derived polysaccharides and legume proteins on the spectral changes observed in vegan yogurts should not be overlooked. Ultimately, it was thought that these molecular changes contribute to characteristic parameters such as gel structure, network organization, and functional properties in vegan yogurts.

## 4. Conclusions

The increasing demand for plant-based products developed as alternatives to fermented dairy products has highlighted the need to enrich the nutritional and functional properties of these products. In this study, the effects of mung bean and black bean protein isolates on the nutritional, structural, and peptide profiles of oat-based vegan yogurts were comprehensively investigated. It has been observed that the addition of different legume protein isolates increases the protein content of oat-based yogurts and makes significant positive contributions to their amino acid composition. In oat-based vegan yogurts, it was found that different legume protein isolates caused changes in structural properties. However, the addition of different legume protein isolates did not result in the formation of new functional groups in FTIR analysis; however, improvements in the spectra suggested that fermentation-induced changes, in addition to protein and polysaccharide components, were influential. Peptide profile analysis revealed numerous short-chain peptides consisting of two to four amino acids, and in silico potential bioactivity predictions showed that these peptides were mostly associated with ACE inhibitor, antioxidant, and antidiabetic activities. It was observed that groups supplemented with mung bean protein isolate formed a richer peptide profile, suggesting that different protein sources may play a decisive role in peptide formation during the fermentation process. In conclusion, when all findings are considered together, it was seen that the use of protein isolate in plant-based yogurt alternatives is not only an additive that increases protein content, but may also make significant contributions to supporting functional and structural properties. Ultimately, a scientific foundation has been established for the design of a new generation of plant-based fermented products with prominent nutraceutical properties. Furthermore, the limited number of replications used in this study restricts the definitive evaluation of the findings and necessitates their confirmation through future studies with a higher number of replications. Furthermore, in addition to comprehensive sensory analyses to assess consumer acceptance of these products, their potential and bioactivity as functional foods should be confirmed through more extensive in vivo and in vitro studies.

## Figures and Tables

**Table 1:** Formulation and coding of oat-based yogurt samples.

Sample	Protein Source	Protein Isolates Level	Description
POY	-	-	Plain oat yogurt (control)
MBPI-2	Mung bean protein isolate	2%	Oat yogurt with 2% mung bean protein isolate
MBPI-4	Mung bean protein isolate	4%	Oat yogurt with 4% mung bean protein isolate
BBPI-2	Black bean protein isolate	2%	Oat yogurt with 2% black bean protein isolate
BBPI-4	Black bean protein isolate	4%	Oat yogurt with 4% black bean protein isolate

**Table 2:** LOD and LOQ values of the amino acid profile (nmol/mL).

Amino Acids	LOD	LOQ	Amino Acids	LOD	LOQ
Arginine	0.05	0.15	Alanine	2.27	6.82
Phenylalanine	0.24	0.73	Asparagine	0.15	0.46
Histidine	0.81	2.43	Aspartic Acid	0.35	1.06
Isoleucine	0.07	0.22	Glycine	3.01	9.03
Lysine	0.03	0.09	Glutamic Acid	0.08	0.24
Leucine	0.07	0.20	Glutamine	0.11	0.32
Methionine	0.14	0.41	Proline	0.22	0.67
Threonine	0.24	0.72	Serine	0.30	0.89
Tryptophan	0.08	0.23	Cystine	0.32	0.97
Valine	1.70	5.11	Tyrosine	1.11	3.34

**Table 3:** Results of physicochemical analyses of oat-based vegan yogurt samples.

	Dry Matter (%)	Protein (%)	Ash (%)	pH
POY	10.03 ± 0.17 ^c^	2.01 ± 0.12 ^e^	0.11 ± 0.00 ^b^	4.40 ± 0.00 ^d^
MBPI-2	11.57 ± 0.58 ^b^	3.60 ± 0.14 ^c^	0.22 ± 0.07 ^a^	4.52 ± 0.03 ^bc^
MBPI-4	13.82 ± 0.16 ^a^	5.20 ± 0.11 ^a^	0.28 ± 0.03 ^a^	4.59 ± 0.01 ^a^
BBPI-2	11.60 ± 0.04 ^b^	2.92 ± 0.21 ^d^	0.25 ± 0.01 ^a^	4.49 ± 0.03 ^c^
BBPI-4	14.14 ± 0.13 ^a^	4.18 ± 0.23 ^b^	0.32 ± 0.04 ^a^	4.56 ± 0.00 ^ab^
Sign.	**	**	*	**
*p*	0.000	0.000	0.021	0.001

^a–e^: Different letters indicate significant differences in the column; Sign.: Significant; **: *p* < 0.01; *: *p* < 0.05; POY: Plain oat yogurt; MBPI-2: Oat yogurt with 2% mung bean protein isolate; MBPI-4: Oat yogurt with 4% mung bean protein isolate; BBPI-2: Oat yogurt with 2% black bean protein isolate; BBPI-4: Oat yogurt with 4% black bean protein isolate.

**Table 4:** Results of microbiological analyses of oat-based vegan yogurt samples (log cfu/g).

	*S. thermophilus*	*L. delbrueckii* subsp. *bulgaricus*	*L. acidophilus*
POY	5.61 ± 0.16 ^c^	5.53 ± 0.10 ^c^	5.69 ± 0.15 ^c^
MBPI-2	6.20 ± 0.49 ^ab^	6.07 ± 0.17 ^ab^	6.05 ± 0.07 ^ab^
MBPI-4	5.94 ± 0.10 ^abc^	5.83 ± 0.07 ^bc^	5.80 ± 0.08 ^bc^
BBPI-2	6.38 ± 0.48 ^a^	6.43 ± 0.30 ^a^	6.11 ± 0.18 ^a^
BBPI-4	5.79 ± 0.07 ^bc^	5.72 ± 0.62 ^bc^	5.58 ± 0.27 ^c^
Sign.	*	*	**
*p*	0.027	0.011	0.002

^a–c^: Different letters indicate significant differences in the column; Sign.: Significant; **: *p* < 0.01; *: *p* < 0.05; POY: Plain oat yogurt; MBPI-2: Oat yogurt with 2% mung bean protein isolate; MBPI-4: Oat yogurt with 4% mung bean protein isolate; BBPI-2: Oat yogurt with 2% black bean protein isolate; BBPI-4: Oat yogurt with 4% black bean protein isolate.

**Table 5:** Results of antioxidant activity analyses of oat-based vegan yogurt samples.

	DPPH Radical Scavenging Activity		ABTS Radical Scavenging Activity	
Samples	Inhibition (%)	µmol TE/g	Inhibition (%)	µmol TE/g
POY	2.39 ± 0.31 ^d^	0.11 ± 0.01 ^d^	8.40 ± 0.64 ^d^	0.40 ± 0.02 ^d^
MBPI-2	12.83 ± 2.16 ^bc^	0.44 ± 0.06 ^bc^	19.85 ± 2.81 ^b^	0.71 ± 0.08 ^b^
MBPI-4	19.93 ± 1.33 ^a^	0.66 ± 0.04 ^a^	28.48 ± 1.62 ^a^	0.94 ± 0.05 ^a^
BBPI-2	10.29 ± 0.21 ^c^	0.36 ± 0.01 ^c^	14.66 ± 1.51 ^c^	0.57 ± 0.04 ^c^
BBPI-4	15.44 ± 1.12 ^b^	0.52 ± 0.04 ^b^	25.35 ± 0.22 ^a^	0.86 ± 0.01 ^a^
Sign.	**	**	**	**
*p*	0.000	0.000	0.000	0.000

^a–d^: Different letters indicate significant differences in the column; Sign.: Significant; **: *p* < 0.01; POY: Plain oat yogurt; MBPI-2: Oat yogurt with 2% mung bean protein isolate; MBPI-4: Oat yogurt with 4% mung bean protein isolate; BBPI-2: Oat yogurt with 2% black bean protein isolate; BBPI-4: Oat yogurt with 4% black bean protein isolate.

**Table 6:** FAA profiles of oat-based vegan yogurt samples (ng/mL).

	POY	MBPI-2	MBPI-4	BBPI-2	BBPI-4	Sign.	*p*
Arginine	23.52 ± 0.51 ^c^	147.35 ± 2.47 ^b^	259.21 ± 8.77 ^a^	139.97 ± 7.70 ^b^	247.70 ± 1.01 ^a^	**	0.000
Phenylalanine	4.06 ± 1.70 ^d^	165.29 ± 12.13 ^b^	252.64 ± 17.23 ^a^	16.89 ± 1.58 ^d^	61.61 ± 5.83 ^c^	**	0.000
Histidine	33.12 ± 2.03 ^c^	52.46 ± 0.23 ^b^	83.37 ± 2.72 ^a^	57.23 ± 2.69 ^b^	81.53 ± 0.81 ^a^	**	0.000
Isoleucine	7.79 ± 3.22 ^c^	113.14 ± 8.61 ^b^	251.01 ± 25.19 ^a^	5.84 ± 0.95 ^c^	15.07 ± 2.33 ^c^	**	0.000
Lysine	11.49 ± 1.08 ^c^	50.48 ± 2.11 ^b^	123.88 ± 13.97 ^a^	10.34 ± 0.69 ^c^	39.54 ± 2.42 ^b^	**	0.000
Leucine	0.49 ± 0.28 ^d^	426.32 ± 8.78 ^b^	777.48 ± 24.27 ^a^	9.75 ± 3.15 ^d^	156.94 ± 0.57 ^c^	**	0.000
Methionine	1.74 ± 0.08 ^d^	161.74 ± 2.87 ^b^	228.78 ± 24.56 ^a^	5.41 ± 1.10 ^d^	88.11 ± 9.63 ^c^	**	0.000
Threonine	7.87 ± 0.76 ^e^	158.80 ± 6.85 ^b^	275.97 ± 0.98 ^a^	24.45 ± 0.22 ^d^	85.70 ± 4.87 ^c^	**	0.000
Tryptophan	5.90 ± 1.96 ^d^	79.14 ± 0.05 ^b^	96.54 ± 6.46 ^a^	59.83 ± 3.54 ^cd^	67.54 ± 1.62 ^c^	**	0.000
Valine	53.38 ± 4.62 ^c^	279.68 ± 28.13 ^b^	525.60 ± 52.11 ^a^	144.68 ± 112.83 ^bc^	125.13 ± 6.23 ^c^	**	0.002
Alanine	21.95 ± 0.09 ^d^	300.71 ± 10.13 ^b^	623.50 ± 50.72 ^a^	46.39 ± 4.49 ^d^	165.50 ± 3.22 ^c^	**	0.000
Asparagine	186.74 ± 24.32 ^c^	312.41 ± 8.13 ^b^	417.53 ± 71.62 ^a^	301.24 ± 40.73 ^b^	396.91 ± 12.69 ^ab^	*	0.011
Aspartic Acid	14.25 ± 1.90 ^a^	119.24 ± 1.82 ^a^	384.73 ± 43.02 ^a^	136.24 ± 5.28 ^a^	561.90 ± 385.42 ^a^	ns	0.109
Glycine	23.67 ± 14.87 ^d^	166.38 ± 5.16 ^b^	350.02 ± 12.21 ^a^	46.77 ± 5.30 ^d^	120.96 ± 13.94 ^c^	**	0.000
Glutamic Acid	11.89 ± 0.06 ^c^	7.97 ± 1.65 ^c^	313.26 ± 71.22 ^a^	6.69 ± 1.00 ^c^	154.71 ± 94.34 ^b^	**	0.007
Glutamine	10.47 ± 0.93 ^c^	87.03 ± 6.45 ^b^	262.18 ± 31.22 ^a^	19.90 ± 4.30 ^c^	14.58 ± 0.09 ^c^	**	0.000
Proline	103.09 ± 3.83 ^c^	242.30 ± 17.77 ^b^	422.86 ± 35.56 ^a^	117.24 ± 12.13 ^c^	126.91 ± 5.20 ^c^	**	0.000
Serine	5.68 ± 7.96 ^d^	134.74 ± 29.10 ^b^	267.73 ± 23.99 ^a^	34.89 ± 2.37 ^cd^	68.01 ± 10.67 ^c^	**	0.000
Cystine	1.08 ± 0.19 ^b^	2.33 ± 0.21 ^a^	1.54 ± 0.25 ^b^	0.99 ± 0.22 ^b^	1.10 ± 0.23 ^b^	**	0.007
Tyrosine	49.69 ± 7.06 ^d^	142.97 ± 6.94 ^b^	265.65 ± 10.53 ^a^	95.47 ± 3.19 ^c^	48.79 ± 1.35 ^d^	**	0.000

^a–e^: Different letters indicate significant differences in the row; Sign.: Significant; ns: not significant; *: *p* < 0.05; **: *p* < 0.01; POY: Plain oat yogurt; MBPI-2: Oat yogurt with 2% mung bean protein isolate; MBPI-4: Oat yogurt with 4% mung bean protein isolate; BBPI-2: Oat yogurt with 2% black bean protein isolate; BBPI-4: Oat yogurt with 4% black bean protein isolate.

**Table 7:** Number of identified peptides in oat-based vegan yogurt.

Samples	Number of Identified Peptides	Number of Antioxidant-Associated Peptides
POY	62	19
MBPI-2	67	21
MBPI-4	81	22
BBPI-2	33	11
BBPI-4	57	16

POY: Plain oat yogurt; MBPI-2: Oat yogurt with 2% mung bean protein isolate; MBPI-4: Oat yogurt with 4% mung bean protein isolate; BBPI-2: Oat yogurt with 2% black bean protein isolate; BBPI-4: Oat yogurt with 4% black bean protein isolate.

## Data Availability

The original contributions presented in the study are included in the article/Supplementary Materials, further inquiries can be directed to the corresponding author.

## Supplementary Materials

The following supporting information can be downloaded at https://www.mdpi.com/article/10.3390/foods15162825/s1, Table S1: Peptide profile and in silico potential bioactivity estimation of oat-based vegan yogurt.
