# Supplementary material for: Oat-Based Vegan Yogurt Alternatives Enriched with Mung Bean and Black Bean Protein Isolates: Nutritional and In Silico Potential Bioactive Peptide Estimation
Source: Foods. 2026 Aug 13;15(16):2825. doi: 10.3390/foods15162825 (PMC13512726; doi:10.3390/foods15162825)
Supplement: Supplementary file 1 [file foods-15-02825-s001.zip › foods-4475986-supplementary.pdf]

Table S1. Peptide profile and in silico potential bioactivity estimation of oat-based vegan yogurt.

Samples	m/z	Sequence	Potential Bioactivity and ID Matched in the BioPEP Database
POY	527,16069	**DDYD**	ACE inhibitor (ID 9072)<br>Peptide regulating ion flow (ID 2749)<br>DPP IV inhibitor (ID 8973)<br>Glutamate carboxypeptidase II inhibitor (ID 10637)
	596,22254	**EMMW**	ACE inhibitor (ID 9085, 9091)<br>Antioxidative peptide (ID 9086)<br>DPP IV inhibitor (ID8690, 8833)<br>Inhibitor of tripeptidyl peptidase II (ID 10796)
	229,1554	**PL**	ACE inhibitor (ID 7513)<br>DPP IV inhibitor (ID 8638)<br>Inhibitor of Xaa-Pro aminopeptidase (ID 10462)<br>Inhibitor of Lactocepin (ID 10463)
	434,16858	**DGGW**	ACE inhibitor (ID 7579, 7616, 7681)<br>Antioxidative peptide (ID 10742)<br>Anti-inflammatory peptide (ID 9865)<br>DPP IV inhibitor (ID 8783, 8787)<br>Inhibitor of tripeptidyl peptidase II (ID 10481)<br>Neprilysin inhibitor (ID 10912)
	434,16823	**DYH**	ACE inhibitor (ID 9072, 9087)<br>Peptide regulating ion flow (ID 2749)<br>Hypotensive peptide (ID 11189)<br>DPP IV inhibitor ID 8937<br>DPP-III inhibitor (ID 9481)<br>Hypouricemic peptide (ID 10715)
	342,14267	**PFG**	ACE inhibitor (ID 7605)<br>DPP IV inhibitor ID 8854<br>DPP-III inhibitor (ID 9505)<br>ACE2 inhibitor (ID 10460)<br>Neprilysin inhibitor (ID 10546)
	433,17085	**FSTG**	ACE inhibitor (ID 7621, 9184)<br>DPP IV inhibitor (ID 8901)
	404,15461	**HEP**	DPP IV inhibitor (ID 8529, 8790)<br>Glutamate carboxypeptidase inhibitor (ID 10624)
	492,18613	**NNMN**	DPP IV inhibitor (ID 8834, 8846, 8847)
	513,17274	**GDHY**	ACE inhibitor (ID 3494, 7620)<br>DPP IV inhibitor (ID 8799)<br>Anti-inflammatory peptide (ID 9869)<br>Neprilysin inhibitor (ID 10910)
	443,20413	**HTW**	Antioxidant peptide (ID 8459)<br>DPP IV inhibitor (ID 8796, 8913)
	474,17573	**HQSC**	DPP IV inhibitor (ID 8877)
	636,27953	**VYWF**	ACE inhibitor (ID 3488, 3492)<br>Antioxidative peptide (ID 11443, 8224)<br>Anti-inflammatory peptide (ID 9868)<br>Hypotensive peptide (ID 11112)<br>DPP IV inhibitor (ID 8692, 8929, 8947)

		DPP-III inhibitor (ID 9509)<br>Inhibitor of tripeptidyl peptidase 2 (ID 10163)
578,21123	**DEEW**	ACE inhibitor (ID 7838)<br>Antithrombotic peptide (ID 3354)<br>Stimulating vasoactive substance release (ID 8329)<br>DPP IV inhibitor (ID 8776)<br>Pancreatic lipase inhibitor (ID 10881)<br>Glutamate carboxypeptidase inhibitor (ID 10622)<br>Glutamate carboxypeptidase II inhibitor (ID 10638, 10643)<br>Inhibitor of citrate lyase deacetylase (ID 10849)<br>Inhibitor of thymidylate synthase (ID 10850)
416,15752	**GSKC**	ACE inhibitor (ID 7607)<br>DPP IV inhibitor (ID 8894)
438,13981	**EMH**	DPP IV inhibitor (ID 8829)
584,20857	**HYEN**	ACE inhibitor (ID 3494, 9078)<br>Anti-inflammatory peptide (ID 9869)<br>DPP IV inhibitor (ID 8799, 8934)<br>Tubulin-tyrosine ligase inhibitor (ID 10606)<br>Glutamate carboxypeptidase inhibitor (ID 10609)<br>Glutamate carboxypeptidase II inhibitor (ID 10610)
509,18748	**DDPY**	Ligand of neurotensin receptor (ID 9657)<br>Antioxidative peptide (ID 10992)<br>Anti-inflammatory peptide (ID 9856)<br>DPP IV inhibitor (ID 8767, 8866)<br>Phospholipase A2 inhibitor (ID 9279)<br>Glutamate carboxypeptidase II inhibitor (ID 10637)
531,17124	**YPDD**	ACE inhibitor (ID 3666)<br>DPP IV inhibitor (ID 8521)<br>Alpha-glucosidase inhibitor (ID 9548)<br>Glutamate carboxypeptidase II inhibitor (ID 10637)
499,14462	**EECP**	Stimulating vasoactive substance release (ID 8329)<br>Glutamate carboxypeptidase inhibitor (ID 10622)<br>Glutamate carboxypeptidase II inhibitor (ID 10643)<br>Inhibitor of citrate lyase deacetylase (ID 10849)<br>Inhibitor of thymidylate synthase (ID 10850)
491,13968	**YDGD**	ACE inhibitor (ID 7620, 7681)<br>DPP IV inhibitor (ID 8933)<br>Neprilysin inhibitor (ID 10910)
657,25135	**CWWY**	ACE inhibitor (ID 10561, 11113)<br>Antioxidative peptide (ID 7898, 10540)<br>DPP IV inhibitor (ID 8683, 8686)
635,26876	**HWRD**	DPP IV inhibitor (ID 8596, 8675, 8798)<br>PPARγ antagonist (ID 11207)
568,20129	**EWDP**	ACE inhibitor (ID 7838)<br>DPP IV inhibitor (ID 8767, 8776, 8930)<br>Pancreatic lipase inhibitor (ID 10881)<br>PPARγ antagonist (ID 11200)
516,2003	**CWHA**	Antioxidative peptide (ID 10540)<br>DPP IV inhibitor (ID 3184, 8931)<br>Alpha-glucosidase inhibitor (ID 10294)<br>Alanine carboxypeptidase inhibitor (ID 10808)

		Antiartherosclerotic peptide (ID 11100)
552,18677	**CFFN**	ACE inhibitor (ID 7751, 10027)<br>DPP IV inhibitor (ID 8778, 10028)<br>Pseudolysin inhibitor (ID 10210)
536,2132	**EERC**	ACE inhibitor (ID 9944)<br>Hypotensive peptide (ID 10925)<br>Neprilysin inhibitor (ID 10909)<br>glutamate carboxypeptidase inhibitor (ID 10622)<br>glutamate carboxypeptidase II inhibitor (ID 10643)<br>citrate lyase deacetylase inhibitor (ID 10849)<br>thymidylate synthase inhibitor (ID 10850)
402,2141	**VHF**	DPP IV inhibitor (ID 8791, 8919)<br>DPP-III inhibitor (ID 9495)
536,21278	**AFNY**	ACE inhibitor (ID 7583, ID 7682)<br>DPP IV inhibitor (ID 8759, 8778, 8853)<br>Inhibitor of tripeptidyl peptidase II (ID 10661)
566,22409	**NTYF**	Antioxidative peptide (ID 8219, 10471)<br>DPP IV inhibitor (ID 8850, 8914, 8935)<br>DPP-III inhibitor (ID 9480)
559,22506	**FWAN**	ACE inhibitor (ID 9089, ID 10719)<br>Development regulating peptide (ID 10721)<br>Peptide activating ubiquitin-mediated proteolysis (ID 4007)<br>DPP IV inhibitor (ID 8528)<br>Neprilysin inhibitor (ID 10720)<br>Hypouricemic peptide (ID 10711)
334,14372	**PMS**	DPP IV inhibitor (ID 8859)
371,22874	**APIA**	ACE inhibitor (ID 7562, ID 7584)<br>DPP IV inhibitor (ID 3177, 8525, 8857)<br>Inhibitor of tripeptidyl peptidase II (ID 10750)<br>Inhibitor of peptidylprolyl isomerase (ID 19863)
388,2554	**AKVA**	ACE inhibitor (ID 10920)<br>DPP IV inhibitor (ID 3172, 8817)<br>Inhibitor of tripeptidyl peptidase 2 (ID 9917)<br>Neprilysin inhibitor (ID 10906)
432,28195	**KAIT**	ACE inhibitor (ID 8193, 7743)<br>Antioxidative (ID 8132)<br>DPP IV inhibitor (ID 3174)<br>DPP-III inhibitor (ID 9511)
415,25559	**PSVI**	DPP IV inhibitor (ID 8862, 8895, 8920)
437,23876	**GTFI**	ACE inhibitor (ID 7612, 8185)<br>Hypotensive peptide (ID 10885)<br>DPP IV inhibitor (ID 8900)<br>DPP-III inhibitor (ID 9486)<br>Renin inhibitor (ID 9471)<br>Pancreatic lipase inhibitor (ID 10880)
354,14716	**GMF**	ACE inhibitor (ID 3385, 7597)<br>DPP IV inhibitor (ID 8827)
565,15865	**EECY**	Glutamate carboxypeptidase inhibitor (ID 10622)<br>Glutamate carboxypeptidase II inhibitor (ID 10643)<br>Inhibitor of citrate lyase deacetylase (ID 10849)<br>Inhibitor of thymidylate synthase (ID 10850)

562,19313	**FDMQ**	ACE inhibitor (ID 9075)<br>Antioxidative peptide (ID 11197)<br>DPP IV inhibitor (ID 8835)
481,26533	**VISY**	ACE inhibitor (ID 7684)<br>DPP IV inhibitor (ID 8897, 8920)
459,28276	**ILDV**	ACE inhibitor (ID 9079)<br>Neuropeptide (ID 10455)<br>DPP IV inhibitor (ID 8802)
525,29182	**NVRH**	ACE inhibitor (ID 7628)<br>DPP IV inhibitor (ID 8594, 8851, 8883)
518,20386	**FLNC**	ACE inhibitor (ID 7832, 11103)<br>Neuropeptide (ID 11101)<br>DPP IV inhibitor (ID 8555, 8823)<br>DPP-III inhibitor (ID 9502)<br>Pseudolysin inhibitor (ID 11213)
440,20622	**HHF**	Antioxidative peptide (ID 3319, 7915)<br>DPP IV inhibitor (ID 8791, 8792)<br>DPP-III inhibitor (ID 9495)
547,33628	**PFKR**	ACE inhibitor (ID 7586, 9211, 10718)<br>Antiviral peptide (ID 11018)<br>DPP IV inhibitor (ID 8814, 8854)<br>DPP-III inhibitor (ID 9505)<br>ACE2 inhibitor (ID 10460)<br>Hypouricemic peptide (ID 10709)
569,31835	**KRPF**	ACE inhibitor (ID 7582, 7586)<br>Immunomodulating (ID 3626)<br>Neuropeptide (ID 10116)<br>DPP IV inhibitor (ID 8518, 8814, 8854)<br>DPP-III inhibitor (ID 9505)<br>ACE2 inhibitor (ID 10460)<br>Neurolysin inhibitor (ID 10171,<br>Neprilysin inhibitor (ID 10918)
613,34428	**KYRF**	ACE inhibitor (ID 3489, 7691)<br>Neuropeptide (ID 9534)<br>Antioxidative peptide (ID 10464)<br>Hypotensive peptide (ID 11190)<br>DPP IV inhibitor (ID 8819, 8444)<br>DPP III inhibitor (ID 9484, 9490)<br>Leucyltransferase inhibitor (ID 10547)
438,24155	**ARIG**	ACE inhibitor (ID 7595, 7742)<br>Neprilysin inhibitor (ID 10907)<br>DPP IV inhibitor (ID 8884)
515,1578	**EEES**	Antioxidative peptide (ID 11335)<br>DPP IV inhibitor (ID 8773)<br>Alpha-glucosidase inhibitor (ID 11334)<br>Glutamate carboxypeptidase inhibitor (ID 10622)<br>Glutamate carboxypeptidase II inhibitor (ID 10643, 10644)<br>Inhibitor of citrate lyase deacetylase (ID 10849)<br>Inhibitor of thymidylate synthase (ID 10850)
597,2686	**TYWQ**	ACE inhibitor (ID 3488)<br>Antioxidative peptide (ID 8219)

		Anti-inflammatory (ID 9868)<br>DPP IV inhibitor (ID 8678, 8914, 8947)<br>PPARγ antagonist (ID 11203)
430,24608	**PWK**	Antioxidative (ID 8190)<br>DPP IV inhibitor (ID 8676, 8865)
595,20412	**HWQC**	DPP IV inhibitor (ID 8678, 8798)<br>PPARγ antagonist (ID 11203)
441,24783	**KFF**	ACE inhibitor (ID 7692, 10027)<br>DPP IV inhibitor (ID 8809, 10028)<br>CaMPDE inhibitor (ID 8249)<br>Renin inhibitor (ID 8248)<br>Pseudolysin inhibitor (ID 10210)
383,21329	**RAH**	ACE inhibitor (ID 7588, 7835)<br>DPP IV inhibitor (ID 8526, 8761)<br>Antioxidative (ID 7886)
539,249	**FPEF**	ACE inhibitor (ID 3502, 9942)<br>DPP IV inhibitor (ID 8506)<br>Alpha-glucosidase inhibitor (ID 9694)<br>DPP III inhibitor (ID 9504)<br>CaMPDE inhibitor (ID 8250)<br>Renin inhibitor (ID 8251)<br>Hypolipidemic peptide (ID 9580)<br>Alcohol dehydrogenase activator (ID 10979)
406,22505	**CKR**	ACE inhibitor (ID 7586)<br>DPP IV inhibitor (ID 8814)
331,21892	**RR**	ACE inhibitor (ID 7741)<br>Antibacterial peptide (ID 10450)<br>Neuropeptide (ID 10454)<br>Hemolytic peptide (ID 10451)<br>Antiviral peptide (ID 10458)<br>DPP IV inhibitor (ID 8889)<br>DPP III inhibitor (ID 9485)<br>Leucyltransferase inhibitor (ID 10457)
375,20165	**WGL**	ACE inhibitor (ID 7599, 7613)<br>Antioxidative peptide (ID 9082)<br>DPP IV inhibitor (ID 8561, 8697)<br>Renin inhibitor (ID 10701)
415,21418	**MHK**	ACE inhibitor (ID 7844)<br>Neuropeptide (ID 10111)<br>DPP IV inhibitor (ID 8829, 9494)
425,21773	**VVW**	ACE inhibitor (ID 3486, 10924, 11400)<br>Antioxidative peptide (ID 8461)<br>DPP IV inhibitor (ID 3183, 8928)<br>Alpha-glucosidase inhibitor (ID 9387)<br>Inhibitor of tripeptidyl peptidase II (ID 19797)<br>Neprilysin inhibitor (ID 19714)<br>Xanthine oxidase inhibitor (ID 11193, 11397)
485,29284	**GPRR**	ACE inhibitor (ID 3537, 7512, 7741, 10903)<br>Antibacterial peptide (ID 10450)<br>Antithrombotic peptide (ID 3047, 3283)<br>Neuropeptide (ID 10454)

			Antioxidative peptide (ID 10927)<br>Hypotensive peptide (ID 10926)<br>Antiviral peptide (ID 10458)<br>DPP IV inhibitor (ID 3169, 8889, 10902)<br>DPP-III inhibitor (ID 9485, 9489)<br>Leucyltransferase inhibitor (ID 10457)<br>Neprilysin inhibitor (ID 10913)
**MBPI-2**	502,27308	**QRAQ**	ACE inhibitor (ID 7588)<br>DPP IV inhibitor (ID 8526)
	431,23293	**IVPC**	ACE inhibitor (ID 7587)<br>DPP IV inhibitor (ID 3181)
	527,16214	**DDYD**	ACE inhibitor (ID 9072)<br>Peptide regulating ion flow (ID 2749)<br>DPP IV inhibitor (ID 8973)<br>Glutamate carboxypeptidase II inhibitor ID10637
	521,22444	**EEFP**	ACE inhibitor (ID 3502, 9942)<br>DPP IV inhibitor (ID 8506)<br>CaMPDE inhibitor (ID 8250)<br>Renin inhibitor (ID 8251)<br>Hypolipidemic (ID 9580)<br>Glutamate carboxypeptidase inhibitor (ID 10622)<br>Glutamate carboxypeptidase II inhibitor (ID 10643)<br>Inhibitor of citrate lyase deacetylase (ID 10849)<br>Inhibitor of thymidylate synthase (ID 10850)
	460,20498	**PDTQ**	ACE inhibitor (ID 7834)<br>DPP IV inhibitor (ID 8908)
	619,30968	**RWVH**	ACE inhibitor (ID 7580, 11195)<br>Antioxidative peptide (ID 8214, 11196)<br>DPP IV inhibitor (ID 8556, 8890, 8919)<br>DPP-III inhibitor (ID 9477)<br>Xanthine oxidase inhibitor (ID 11194)
	372,19007	**IAF**	ACE inhibitor (ID 7562, 7583, 10076)<br>DPP IV inhibitor (ID 8525, 8759)<br>HMG-CoA reductase inhibitor (ID 10235)<br>Inhibitor of tripeptidyl peptidase II (ID 10661)
	357,2511	**LAR**	ACE inhibitor (ID 7585, 7742)<br>DPP IV inhibitor (ID 3175)<br>DPP-III inhibitor (ID 9499)<br>Neprilysin inhibitor (ID 10907)
	357,25085	**IPK**	ACE inhibitor (ID 7581)<br>DPP IV inhibitor (ID 8501, 8858)<br>Antioxidative peptide (ID 10771)
	480,2585	**HQVP**	ACE inhibitor (ID 7587)<br>DPP IV inhibitor (ID 3181, 8879)
	281,15037	**YV**	ACE inhibitor (ID 9077)<br>DPP IV inhibitor (ID 8946)
	608,31997	**KWQF**	ACE inhibitor (ID 3386)<br>DPP IV inhibitor (ID 8678, 8818, 8870)<br>Renin inhibitor (ID 9431)<br>PPARγ antagonist (ID 11203)
	401,28845	**IRI**	ACE inhibitor (ID 3258)

		Antioxidative peptide (ID 8215)<br>DPP IV inhibitor (ID 8806, 8884)<br>CaMPDE inhibitor (ID 8247)<br>Renin inhibitor (ID 8246)
683,30396	**RHYW**	ACE inhibitor (ID 3488, 3494)<br>Antioxidative peptide (ID 8070)<br>Anti-inflammatory peptide (ID 9868, 9869)<br>DPP IV inhibitor (ID 8799, 8883, 8947)
488,25239	**MIPQ**	ACE inhibitor (ID 7581, 7837)<br>DPP IV inhibitor (ID 8501, 8830, 8861)
366,21467	**PLH**	ACE inhibitor (ID 7513)<br>Neuropeptide (ID 11104)<br>Antioxidative peptide (ID 3305)<br>DPP IV inhibitor (ID 8638, 8820)<br>Inhibitor of Xaa-Pro aminopeptidase (ID 10462)<br>Inhibitor of Lactocepin (ID 10463)
333,19249	**KW**	ACE inhibitor (ID 3386)<br>DPP IV inhibitor (ID 8818)
456,29457	**IAPR**	ACE inhibitor (ID 3537, 7562, 7584, 7819)<br>DPP IV inhibitor (ID 3177, 8525)<br>DPP-III inhibitor (ID 9489)<br>Inhibitor of tripeptidyl peptidase II (ID 10750)<br>Inhibitor of peptidylprolyl isomerase (ID 10863)
294,14603	**AGF**	ACE inhibitor (ID 7591, 7600)<br>DPP IV inhibitor (ID 8760, 8782)<br>DPP-III inhibitor (ID 9488)<br>Tripeptidyl peptidase II inhibitor (ID 10584)<br>Acylaminoacyl peptidase inhibitor (ID 10583)
365,18394	**MSK**	DPP IV inhibitor (ID 8894)
231,17086	**LV**	DPP IV inhibitor (ID 8825)
336,19082	**VVP**	ACE inhibitor (ID 7587, 10924)<br>DPP IV inhibitor (ID 3181, 3183)<br>Inhibitor of tripeptidyl peptidase II (ID 10797)<br>Neprilysin inhibitor (ID 10916)
532,20503	**DDPW**	Antioxidative (ID 8190)<br>DPP IV inhibitor (ID 8767, 8865)<br>Glutamate carboxypeptidase II inhibitor (ID 10637)
444,28337	**KTPV**	ACE inhibitor (ID 9073)<br>DPP IV inhibitor (ID 8503, 8816, 8864)<br>Hypouricemic peptide (ID 10747)
548,18093	**ECFQ**	ACE inhibitor (ID 7751, 9076)<br>DPP IV inhibitor (ID 8779)
510,23652	**KYPC**	ACE inhibitor (ID 3666, 7691)<br>Antioxidative (ID 10464)<br>Hypotensive peptide (ID 11190)<br>DPP IV inhibitor (ID 8521, 8819)<br>Alpha-glucosidase inhibitor (ID 9548)
336,18949	**VPV**	ACE inhibitor (ID 7587)<br>DPP IV inhibitor (ID 3181, 8864)<br>Antidiabetic peptide (ID 11008)
474,17719	**HCNT**	Antioxidative peptide (ID 10257)

		Anti-inflammatory peptide (ID 10349)<br>DPP IV inhibitor (ID 8850)
474,17606	**HQSC**	DPP IV inhibitor ID 8877
295,16643	**YI**	ACE inhibitor (ID 11114)<br>Neuropeptide (ID 11115)<br>DPP IV inhibitor (ID 8938)<br>DPP-III inhibitor (ID 9510)
263,14012	**PF**	DPP IV inhibitor (ID 8854)<br>DPP-III inhibitor (ID 9505)<br>ACE2 inhibitor (ID 10460)
265,15571	**VF**	ACE inhibitor (ID 3384)<br>DPP IV inhibitor (ID 8917)<br>Calpain 1 inhibitor (ID9918)<br>Inhibitor of tripeptidyl peptidase II (ID 9920)<br>Inhibitor of neprilysin-2 (ID 10095)
416,15797	**GSKC**	ACE inhibitor (ID 7607)<br>DPP IV inhibitor (ID 8894)
416,1578	**EMH**	DPP IV inhibitor (ID 8829)
435,2736	**IRF**	ACE inhibitor (ID 3258, 3489)<br>Antioxidative (ID 8215)<br>DPP IV inhibitor (ID 8806)<br>DPP III inhibitor (ID 9490)<br>CaMPDE inhibitor (ID 8247)<br>Renin inhibitor (ID 8246)<br>Leucyltransferase inhibitor (ID 10547)
359,26611	**KVL**	DPP IV inhibitor (ID 8817, 8922)
328,22449	**PVI**	DPP IV inhibitor (ID 8864, 8920)
391,20215	**CVAV**	ACE inhibitor (ID 7635, 8951)<br>DPP IV inhibitor (ID 3172, 8764)<br>Inhibitor of tripeptidyl peptidase 2 (ID 9917)
587,29932	**PVWW**	ACE inhibitor (ID 3486, 10561)<br>Antioxidative peptide (ID 8461)<br>DPP IV inhibitor (ID 8686, 8864, 8928)<br>Alpha-glucosidase inhibitor (ID 9387)<br>Hypouricemic peptide (ID 10714)<br>Xanthine oxidase inhibitor (ID 11193)
371,22989	**APIA**	ACE inhibitor (ID 7562, ID 7584)<br>DPP IV inhibitor (ID 3177, 8525, 8857)<br>Inhibitor of tripeptidyl peptidase II (ID 10750)<br>Inhibitor of peptidylprolyl isomerase (ID 19863)
388,25663	**AKVA**	ACE inhibitor (ID 10920)<br>DPP IV inhibitor (ID 3172, 8817)<br>Inhibitor of tripeptidyl peptidase 2 (ID 9917)<br>Neprilysin inhibitor (ID 10906)
245,18707	**LL**	DPP IV inhibitor (ID 3182)<br>Inhibitor of Lactocepin (ID 10619)<br>Inhibitor of cytosol alanyl aminopeptidase (ID 10615)
279,17094	**FL**	ACE inhibitor (ID 11103)<br>Neuropeptide (ID 11101)<br>DPP IV inhibitor (ID 8555)<br>DPP-III inhibitor (ID 9502)

		Pseudolysin inhibitor (ID 11213)
432,28161	**KAIT**	ACE inhibitor (ID 8193, 7743)<br>Antioxidative (ID 8132)<br>DPP IV inhibitor (ID 3174)<br>DPP-III inhibitor (ID 9511)
415,25516	**PSVI**	DPP IV inhibitor (ID 8862, 8895, 8920)
437,23833	**GTFI**	ACE inhibitor (ID 7612, 8185)<br>Hypotensive peptide (ID 10885)<br>DPP IV inhibitor (ID 8900)<br>DPP-III inhibitor (ID 9486)<br>Renin inhibitor (ID 9471)<br>Pancreatic lipase inhibitor (ID 10880)
616,3129	**LIWY**	ACE inhibitor (ID 7544, 11113)<br>Antioxidative peptide (ID 7898)<br>Hypotensive peptide (ID 11192)<br>DPP IV inhibitor (ID 8683, 8807, 8821)
459,28232	**ILDV**	ACE inhibitor (ID 9079)<br>Neuropeptide (ID 10455)<br>DPP IV inhibitor (ID 8802)
481,26518	**VISY**	ACE inhibitor (ID 7684)<br>DPP IV inhibitor (ID 8897, 8920)
547,33526	**PFRK**	ACE inhibitor (ID 7592)<br>DPP IV inhibitor (ID 8780, 8854, 8885)<br>DPP III inhibitor (ID 9501, 9505)<br>ACE2 inhibitor (ID 10460)<br>Hypouricemic peptide (ID 10710)
569,31826	**KPRF**	ACE inhibitor (ID 3489, 3537, 7810)<br>Antioxidative peptide (ID 8218)<br>DPP IV inhibitor (ID 8519)<br>DPP III inhibitor (ID 9489, 9490)<br>Leucyltransferase inhibitor (ID 10547)
613,34502	**KYRF**	ACE inhibitor (ID 3489, 7691)<br>Neuropeptide (ID 9534)<br>Antioxidative peptide (ID 10464)<br>Hypotensive peptide (ID 11190)<br>DPP IV inhibitor (ID 8819, 8444)<br>DPP III inhibitor (ID 9484, 9490)<br>Leucyltransferase inhibitor (ID 10547)
515,15813	**TDEE**	Antithrombotic peptide (ID 3354)<br>Stimulating vasoactive substance release (ID 8329)<br>DPP IV inhibitor (ID 8898)<br>Glutamate carboxypeptidase inhibitor (ID 10622)<br>Glutamate carboxypeptidase II inhibitor (ID 10638, 10643)<br>Inhibitor of citrate lyase deacetylase (ID 10849)<br>Inhibitor of thymidylate synthase (ID 10850)
580,2987	**HVRF**	ACE inhibitor (ID 3489, 7628, 10692)<br>DPP IV inhibitor (ID 8594, 8797)<br>DPP III inhibitor (ID 9490)<br>Leucyltransferase inhibitor (ID 10547)<br>ACE 2 activator (ID 10684)
575,28617	**FFVY**	ACE inhibitor (ID 3492, 10027)

		Antioxidative peptide (ID 8224)<br>Hypotensive peptide (ID 11112)<br>DPP IV inhibitor (ID 8929, 10028)<br>DPP-III inhibitor (ID 9509)<br>Inhibitor of tripeptidyl peptidase 2 (ID 10163)<br>Pseudolysin inhibitor (ID 10210)
358,27143	**ILL**	ACE inhibitor (ID 9079)<br>Neuropeptide (ID 10455)<br>DPP IV inhibitor (ID 3182, 8802)<br>Inhibitor of Lactocepin (ID 10619)<br>Inhibitor of cytosol alanyl aminopeptidase (ID 10615)
447,22689	**MSPL**	ACE inhibitor (ID 7513)<br>DPP IV inhibitor (ID 8505, 8638)<br>Inhibitor of Xaa-Pro aminopeptidase (ID 10462)<br>Inhibitor of Lactocepin (ID 10463)<br>Inhibitor of peptidylprolyl isomerase (ID 11279)
469,20897	**WTY**	ACE inhibitor (ID 10991)<br>Antioxidative peptide (ID 8219)<br>DPP IV inhibitor (ID 8685, 8914)
447,22599	**PPYA**	ACE inhibitor (ID 7589, 7836)<br>Antioxidative peptide (ID 10470, 10992)<br>Anti-inflammatory peptide (ID 9856)<br>DPP IV inhibitor (ID 3170, 8866, 8932)<br>Alpha-glucosidase inhibitor (ID 9651)<br>Renin inhibitor (ID 9433)<br>Phospholipase A2 inhibitor (ID 9279)<br>Tubulin-tyrosine ligase inhibitor (ID 10605)<br>Alanine carboxypeptidase inhibitor (ID 10608)
602,37923	**KRWI**	ACE inhibitor (ID 7580, 7586)<br>Antioxidative peptide (ID 8075, 8214)<br>DPP IV inhibitor (ID 8679, 8814, 8890)<br>DPP-III inhibitor (ID 9477)
443,25018	**IPVD**	ACE inhibitor (ID 7581)<br>DPP IV inhibitor (ID 8501, 8864, 8915)
331,21875	**RR**	ACE inhibitor (ID 7741)<br>Antibacterial peptide (ID 10450)<br>Neuropeptide (ID 10454)<br>Hemolytic peptide (ID 10451)<br>Antiviral peptide (ID 10458)<br>DPP IV inhibitor (ID 8889)<br>DPP III inhibitor (ID 9485)<br>Leucyltransferase inhibitor (ID 10457)
425,26399	**HRI**	DPP IV inhibitor (ID 8794, 8884)
375,20134	**WGL**	ACE inhibitor (ID 7599, 7613)<br>Antioxidative peptide (ID 9082)<br>DPP IV inhibitor (ID 8561, 8697)<br>Renin inhibitor (ID 10701)
425,2178	**VVW**	ACE inhibitor (ID 3486, 10924, 11400)<br>Antioxidative peptide (ID 8461)<br>DPP IV inhibitor (ID 3183, 8928)<br>Alpha-glucosidase inhibitor (ID 9387)

			Inhibitor of tripeptidyl peptidase II (ID 19797)<br>Neprilysin inhibitor (ID 19714)<br>Xanthine oxidase inhibitor (ID 11193, 11397)
	485,29323	**GPRR**	ACE inhibitor (ID 3537, 7512, 7741, 10903)<br>Antibacterial peptide (ID 10450)<br>Antithrombotic peptide (ID 3047, 3283)<br>Neuropeptide (ID 10454)<br>Antioxidative peptide (ID 10927)<br>Hypotensive peptide (ID 10926)<br>Antiviral peptide (ID 10458)<br>DPP IV inhibitor (ID 3169, 8889, 10902)<br>DPP-III inhibitor (ID 9485, 9489)<br>Leucyltransferase inhibitor (ID 10457)<br>Neprilysin inhibitor (ID 10913)
	479,26565	**CKKT**	ACE inhibitor (ID 10921)<br>DPP IV inhibitor (ID 8813, 8816)<br>Neprilysin inhibitor (ID 10915)
**MBPI-4**	527,16086	**DDYD**	ACE inhibitor (ID 9072)<br>peptide regulating ion flow (ID 2749)<br>DPP IV inhibitor (ID 8973)<br>Glutamate carboxypeptidase II inhibitor (ID 10637)
	487,18209	**EVMS**	ACE inhibitor (ID 7828, 9882)<br>DPP IV inhibitor (ID 8775, 8923)<br>Inhibitor of tripeptidyl peptidase II (ID 10839)
	619,30879	**RWVH**	ACE inhibitor (ID 7580, 11195)<br>Antioxidative peptide (ID 8214, 11196)<br>DPP IV inhibitor (ID 8556, 8890, 8919)<br>DPP-III inhibitor (ID 9477)<br>Xanthine oxidase inhibitor (ID 11194)
	229,15504	**PL**	ACE inhibitor (ID 7513)<br>DPP IV inhibitor (ID 8638)<br>Inhibitor of Xaa-Pro aminopeptidase (ID 10462)<br>Inhibitor of Lactocepin (ID 10463)
	372,18932	**IAF**	ACE inhibitor (ID 7562, 7583, 10076)<br>DPP IV inhibitor (ID 8525, 8759)<br>HMG-CoA reductase inhibitor (ID 10235)<br>Inhibitor of tripeptidyl peptidase II (ID 10661)
	359,24114	**LAR**	ACE inhibitor (ID 7585, 7742)<br>DPP IV inhibitor (ID 3175)<br>DPP-III inhibitor (ID 9499)<br>Neprilysin inhibitor (ID 10907)
	310,1412	**SFG**	ACE inhibitor (ID 7605, 7685)<br>DPP IV inhibitor ID 8891<br>Renin inhibitor (ID 9432)<br>Neprilysin inhibitor (ID 10546)
	401,28843	**IRI**	ACE inhibitor (ID 3258)<br>Antioxidative peptide (ID 8215)<br>DPP IV inhibitor (ID 8806, 8884)<br>CaMPDE inhibitor (ID 8247)<br>Renin inhibitor (ID 8246)
	389,21689	**WAI**	ACE inhibitor (ID 8193, 9089)

		DPP IV inhibitor (ID 8528)
487,31323	**KQLT**	Antioxidative peptide (ID 10749)<br>DPP IV inhibitor (ID 8824, 8874)<br>Hypouricemic peptide (ID 10741)
480,25923	**FKW**	ACE inhibitor (ID 3386, 10718)<br>DPP IV inhibitor (ID 8818)<br>Hypouricemic peptide (ID 10709)
385,25679	**PRI**	ACE inhibitor (ID 3537, 9755)<br>DPP IV inhibitor (ID 8884)<br>DPP-III inhibitor (ID 9489)
480,2584	**HQVP**	ACE inhibitor (ID 7587)<br>DPP IV inhibitor (ID 3181, 8879)
608,31976	**KWQF**	ACE inhibitor (ID 3386)<br>DPP IV inhibitor (ID 8678, 8818, 8870)<br>Renin inhibitor (ID 9431)<br>PPARγ antagonist (ID 11203)
281,15046	**YV**	ACE inhibitor (ID 9077)<br>DPP IV inhibitor (ID 8946)
423,23601	**HGLP**	ACE inhibitor (ID 7509, 7599, 7614, 10092)<br>DPP IV inhibitor (ID 3180, 8561)<br>Inhibitor of Lactocepin (ID 10620)
231,17078	**LV**	DPP IV inhibitor (ID 8825)
488,25734	**NQRA**	ACE inhibitor (ID 7588)<br>DPP IV inhibitor (ID 8526, 8848)
510,23263	**WSFA**	ACE inhibitor (ID 7685)<br>DPP IV inhibitor (ID 3176, 8687, 8891)<br>Alpha-glucosidase inhibitor (ID 10297)<br>DPP-III inhibitor (ID 9500)<br>Renin inhibitor (ID 9432)<br>Alanine carboxypeptidase inhibitor (ID 10809)
683,30296	**RHYW**	ACE inhibitor (ID 3488, 3494)<br>Antioxidative peptide (ID 8070)<br>Anti-inflammatory peptide (ID 9868, 9869)<br>DPP IV inhibitor (ID 8799, 8883, 8947)
333,18652	**SRA**	ACE inhibitor (ID 7588)<br>DPP IV inhibitor (ID 8526)
551,29056	**KRPE**	ACE inhibitor (ID 7582, 7586)<br>Immunomodulating (ID 3626)<br>Neuropeptide (ID 10116)<br>DPP IV inhibitor (ID 8518, 8814)<br>Alpha-glucosidase inhibitor (ID 9694)<br>DPP-III inhibitor (ID 9504)<br>Neurolysin inhibitor (ID 10171,<br>Neprilysin inhibitor (10918)
366,21299	**PLH**	ACE inhibitor (ID 7513)<br>Neuropeptide (ID 11104)<br>Antioxidative peptide (ID 3305)<br>DPP IV inhibitor (ID 8638, 8820)<br>Inhibitor of Xaa-Pro aminopeptidase (ID 10462)<br>Inhibitor of Lactocepin (ID 10463)
333,19289	**KW**	ACE inhibitor (ID 3386)

		DPP IV inhibitor (ID 8818)
551,29343	**TRQF**	DPP IV inhibitor (ID 8870, 8909)<br>Renin inhibitor (ID 9431)
551,29541	**IFHL**	ACE inhibitor (ID 7593, 7602)<br>Antioxidative peptide (ID 3317)<br>DPP IV inhibitor (ID 8557)<br>DPP-III inhibitor (ID 9493)<br>Hypouricemic peptide (ID 10708)
456,29513	**IAPR**	ACE inhibitor (ID 3537, 7562, 7584, 7819)<br>DPP IV inhibitor (ID 3177, 8525)<br>DPP-III inhibitor (ID 9489)<br>Inhibitor of tripeptidyl peptidase II (ID 10750)<br>Inhibitor of peptidylprolyl isomerase (ID 10863)
294,146	**AGF**	ACE inhibitor (ID 7591, 7600)<br>DPP IV inhibitor (ID 8760, 8782)<br>DPP-III inhibitor (ID 9488)<br>Tripeptidyl peptidase II inhibitor (ID 10584)<br>Acylaminoacyl peptidase inhibitor (ID 10583)
365,1838	**MSK**	DPP IV inhibitor (ID 8894)
345,14576	**YY**	ACE inhibitor (ID 9946)<br>DPP IV inhibitor (ID 8948)<br>DPP-III inhibitor (ID 9476)<br>Tubulin-tyrosine ligase inhibitor (ID 10607)
365,18296	**AAGF**	ACE inhibitor (ID 7590, 7591, 7600)<br>Antibacterial peptide (ID 10663)<br>Hypotensive peptide (ID 10141)<br>DPP IV inhibitor (ID 8637, 8760, 8782)<br>DPP-III inhibitor (ID 9488)<br>Tripeptidyl peptidase II inhibitor (ID 10584)<br>Inhibitor of tripeptidyl peptidase II (ID 10659)<br>Acylaminoacyl peptidase inhibitor (ID 10583)<br>Inhibitor of cytosol alanyl aminopeptidase (ID 10611)<br>Inhibitor of D-Ala-D-Ala dipeptidase (ID 10664)
548,17923	**ECFQ**	ACE inhibitor (ID 7751, 9076)<br>DPP IV inhibitor (ID 8779)
510,2339	**TWGF**	ACE inhibitor (ID 7591, 7613)<br>Antioxidative peptide (ID 8459, 9082)<br>DPP IV inhibitor (ID 8697, 8782, 8913)<br>DPP-III inhibitor (ID 9488)<br>Renin inhibitor (ID 10701)<br>Tripeptidyl peptidase II inhibitor (ID 10584)<br>Acylaminoacyl peptidase inhibitor (ID 10583)
510,23425	**MIPQ**	ACE inhibitor (ID 7581, 7837)<br>DPP IV inhibitor (ID 8501, 8830, 8861)
474,17677	**HCNT**	Antioxidative peptide (ID 10257)<br>Anti-inflammatory peptide (ID 10349)<br>DPP IV inhibitor (ID 8850)
395,22639	**AVAI**	ACE inhibitor (ID 8193, 8951)<br>DPP IV inhibitor (ID 3172, 8764)<br>Inhibitor of tripeptidyl peptidase 2 (ID 9917)
395,22942	**FTK**	DPP IV inhibitor (ID 8904)

		Renin inhibitor (ID 2835)
606,3038	**RYVF**	ACE inhibitor (ID 3380, 3384, 9077)<br>Antioxidative peptide (ID 10051)<br>DPP IV inhibitor (ID 8917, 8946)<br>Calpain 1 inhibitor (ID 9918)<br>Inhibitor of tripeptidyl peptidase II (ID 9920)<br>Inhibitor of neprilysin-2 (ID 10095)
265,15528	**VF**	ACE inhibitor (ID 3384)<br>DPP IV inhibitor (ID 8917)<br>Calpain 1 inhibitor (ID9918)<br>Inhibitor of tripeptidyl peptidase II (ID 9920)<br>Inhibitor of neprilysin-2 (ID 10095)
416,15745	**GSKC**	ACE inhibitor (ID 7607)<br>DPP IV inhibitor (ID 8894)
416,15788	**EMH**	DPP IV inhibitor (ID 8829)
435,27346	**IRF**	ACE inhibitor (ID 3258, 3489)<br>Antioxidative (ID 8215)<br>DPP IV inhibitor (ID 8806)<br>DPP III inhibitor (ID 9490)<br>CaMPDE inhibitor (ID 8247)<br>Renin inhibitor (ID 8246)<br>Leucyltransferase inhibitor (ID 10547)
359,26574	**KVL**	DPP IV inhibitor (ID 8817, 8922)
519,25737	**FFAL**	ACE inhibitor (ID 7823, 10027)<br>DPP IV inhibitor (ID 3176, 8559, 10028)<br>DPP III inhibitor (ID 9500)<br>Pseudolysin inhibitor (ID 10210)<br>Alanine carboxypeptidase inhibitor (ID 10808)
519,25721	**QIMQ**	DPP IV inhibitor (ID 8803, 8835, 8873)
292,13049	**WS**	DPP IV inhibitor (ID 8637)<br>Alpha-glucosidase inhibitor (ID 10297)
487,32566	**KQLV**	DPP IV inhibitor (ID 8321, 8825, 8874)
411,26224	**PVVP**	ACE inhibitor (ID 7587, 10294)<br>DPP IV inhibitor (ID 3181, 3183, 8864)<br>Inhibitor of tripeptidyl peptidase II (ID 10797)<br>Neprilysin inhibitor (ID 10916)
357,21408	**APIG**	ACE inhibitor (ID 7584, 7595)<br>DPP IV inhibitor (ID 3177, 8857)<br>Inhibitor of tripeptidyl peptidase II (ID 10750)<br>Inhibitor of peptidylprolyl isomerase (ID 10863)
328,22437	**PVI**	DPP IV inhibitor (ID 8864, 8920)
391,20237	**CVAV**	ACE inhibitor (ID 7635, 8951)<br>DPP IV inhibitor (ID 3172, 8764)<br>Inhibitor of tripeptidyl peptidase 2 (ID 9917)
366,20259	**ISF**	ACE inhibitor (ID 7685)<br>DPP IV inhibitor (ID 8891)<br>Renin inhibitor (ID 9432)
359,23034	**AVLG**	ACE inhibitor (ID 7619, 8951, 9060)<br>DPP IV inhibitor (ID 8764, 8922)
587,2994	**PVWW**	ACE inhibitor (ID 3486, 10561)<br>Antioxidative peptide (ID 8461)

		DPP IV inhibitor (ID 8686, 8864, 8928)<br>Alpha-glucosidase inhibitor (ID 9387)<br>Hypouricemic peptide (ID 10714)<br>Xanthine oxidase inhibitor (ID 11193)
371,22965	**APIA**	ACE inhibitor (ID 7562, ID 7584)<br>DPP IV inhibitor (ID 3177, 8525, 8857)<br>Inhibitor of tripeptidyl peptidase II (ID 10750)<br>Inhibitor of peptidylprolyl isomerase (ID 19863)
388,25603	**AKVA**	ACE inhibitor (ID 10920)<br>DPP IV inhibitor (ID 3172, 8817)<br>Inhibitor of tripeptidyl peptidase 2 (ID 9917)<br>Neprilysin inhibitor (ID 10906)
441,21596	**PPPM**	ACE inhibitor (ID 7836, 9172)<br>DPP IV inhibitor ID 3170, 8859)<br>Alpha-glucosidase inhibitor (ID 9651)
279,17141	**FL**	ACE inhibitor (ID 11103)<br>Neuropeptide (ID 11101)<br>DPP IV inhibitor (ID 8555)<br>DPP-III inhibitor (ID 9502)<br>Pseudolysin inhibitor (ID 11213)
432,28188	**KAIT**	ACE inhibitor (ID 8193, 7743)<br>Antioxidative (ID 8132)<br>DPP IV inhibitor (ID 3174)<br>DPP-III inhibitor (ID 9511)
415,25596	**PSVI**	DPP IV inhibitor (ID 8862, 8895, 8920)
437,23848	**GTFI**	ACE inhibitor (ID 7612, 8185)<br>Hypotensive peptide (ID 10885)<br>DPP IV inhibitor (ID 8900)<br>DPP-III inhibitor (ID 9486)<br>Renin inhibitor (ID 9471)<br>Pancreatic lipase inhibitor (ID 10880)
593,29673	**YILY**	ACE inhibitor (ID 3381, 9079, 11114)<br>Neuropeptide (ID 10455, 11115)<br>Antioxidative peptide (ID 7872)<br>DPP IV inhibitor (ID 8802, 8938)<br>DPP III inhibitor (ID 9510)<br>Renin inhibitor (ID 9470)
616,31228	**LIWY**	ACE inhibitor (ID 7544, 11113)<br>Antioxidative peptide (ID 7898)<br>Hypotensive peptide (ID 11192)<br>DPP IV inhibitor (ID 8683, 8807, 8821)
459,28238	**ILDV**	ACE inhibitor (ID 9079)<br>Neuropeptide (ID 10455)<br>DPP IV inhibitor (ID 8802)
416,22993	**FLH**	ACE inhibitor (ID 11103, 9079, 11114)<br>Neuropeptide (ID 11101, 11104)<br>Antioxidative peptide (ID 3305)<br>DPP IV inhibitor (ID 8555, 8820)<br>DPP III inhibitor (ID 9502)<br>Pseudolysin inhibitor (ID 11213)
487,28966	**LNQI**	ACE inhibitor (ID 7832)

		DPP IV inhibitor (ID 8823, 8848, 8873)
502,17377	**GMWS**	ACE inhibitor (ID 7597, 9091)<br>DPP IV inhibitor ID 8687, 8690)<br>Alpha-glucosidase inhibitör (ID 10297)
580,29817	**HVRF**	ACE inhibitor (ID 3489, 7628, 10692)<br>DPP IV inhibitor (ID 8594, 8797)<br>DPP III inhibitor (ID 9490)<br>Leucyltransferase inhibitor (ID 10547)<br>ACE 2 activator (ID 10684)
358,27114	**ILL**	ACE inhibitor (ID 9079)<br>Neuropeptide (ID 10455)<br>DPP IV inhibitor (ID 3182, 8802)<br>Inhibitor of Lactocepin (ID 10619)<br>Inhibitor of cytosol alanyl aminopeptidase (ID 10615)
430,2464	**PWK**	Antioxidative (ID 8190)<br>DPP IV inhibitor (ID 8676, 8865)
597,26782	**FFVY**	ACE inhibitor (ID 3492, 10027)<br>antioxidative peptide (ID 8224)<br>Hypotensive peptide (ID 11112)<br>DPP IV inhibitor (ID 8929, 10028)<br>DPP-III inhibitor (ID 9509)<br>Inhibitor of tripeptidyl peptidase 2 (ID 10163)<br>Pseudolysin inhibitor (ID 10210)
352,16698	**WF**	Antioxidative peptide (ID 11443)<br>DPP IV inhibitor (ID 8692)
469,20866	**WTY**	ACE inhibitor (ID 10991)<br>Antioxidative peptide (ID 8219)<br>DPP IV inhibitor (ID 8685, 8914)
447,22616	**MSPL**	ACE inhibitor (ID 7513)<br>DPP IV inhibitor (ID 8505, 8638)<br>Inhibitor of Xaa-Pro aminopeptidase (ID 10462)<br>Inhibitor of Lactocepin (ID 10463)<br>Inhibitor of peptidylprolyl isomerase (ID 11279)
443,26309	**KQPA**	ACE inhibitor (ID 9566, 10923)<br>DPP IV inhibitor (ID 3179, 8532)
369,22612	**PRP**	ACE inhibitor (ID 3537, 7582, 10551)<br>Neuropeptide (ID 10116)<br>DPP IV inhibitor (ID 8518)<br>DPP-III inhibitor (ID 9489)<br>Neurolysin inhibitor (ID 10171,<br>Neprilysin inhibitor (ID10918)
441,23101	**SISL**	ACE inhibitor (ID 11318)<br>DPP IV inhibitor (ID 8560, 8893)
375,20113	**WGL**	ACE inhibitor (ID 7599, 7613)<br>Antioxidative peptide (ID 9082)<br>DPP IV inhibitor (ID 8561, 8697)<br>Renin inhibitor (ID 10701)
415,21415	**MHK**	ACE inhibitor (ID 7844)<br>Neuropeptide (ID 10111)<br>DPP IV inhibitor (ID 8829, 9494)
425,21725	**VVW**	ACE inhibitor (ID 3486, 10924, 11400)

			Antioxidative peptide (ID 8461)<br>DPP IV inhibitor (ID 3183, 8928)<br>Alpha-glucosidase inhibitor (ID 9387)<br>Inhibitor of tripeptidyl peptidase II (ID 19797)<br>Neprilysin inhibitor (ID 19714)<br>Xanthine oxidase inhibitor (ID 11193, 11397)
	485,29312	**GPRR**	ACE inhibitor (ID 3537, 7512, 7741, 10903)<br>Antibacterial peptide (ID 10450)<br>Antithrombotic peptide (ID 3047, 3283)<br>Neuropeptide (ID 10454)<br>Antioxidative peptide (ID 10927)<br>Hypotensive peptide (ID 10926)<br>Antiviral peptide (ID 10458)<br>DPP IV inhibitor (ID 3169, 8889, 10902)<br>DPP-III inhibitor (ID 9485, 9489)<br>Leucyltransferase inhibitor (ID 10457)<br>Neprilysin inhibitor (ID 10913)
**BBPI-2**	527,15916	**CGYY**	ACE inhibitor (ID 3532, 9946)<br>Opioid (ID 3786)<br>Anticancer (ID 10468)<br>Antioxidative peptide (ID 7867)<br>DPP IV inhibitor (ID 8788, 8948)<br>DPP-III inhibitor (ID 9476)<br>Inhibitor of tripeptidyl peptidase II (ID 10754)<br>Tubulin-tyrosine ligase inhibitor (ID 10607)
	365,12923	**DGSS**	ACE inhibitor (ID 7607, 7681)
	229,1555	**PL**	ACE inhibitor (ID 7513)<br>DPP IV inhibitor ID 8638<br>Inhibitor of Xaa-Pro aminopeptidase (ID 10462)<br>Inhibitor of Lactocepin (ID 10463)
	474,17522	**HQSC**	DPP IV inhibitor ID 8877
	416,1569	**GSKC**	ACE inhibitor (ID 7607)<br>DPP IV inhibitor (ID 8894)
	352,08096	**CDD**	Glutamate carboxypeptidase II inhibitor (ID 10637)
	493,13691	**DAYC**	ACE inhibitor (ID 3563, 7606)<br>Antioxidative (ID 7866)<br>DPP IV inhibitor (ID 8765)<br>DPP-III inhibitor (ID 9492)<br>Tubulin-tyrosine ligase inhibitor (ID 10604)<br>Glutamate carboxypeptidase II inhibitor (ID 10636)
	479,12132	**DYGC**	ACE inhibitor (ID 3553, 9072)<br>Antioxidative (ID 10539)<br>DPP IV inhibitor (ID 8936)<br>DPP-III inhibitor (ID 9508)<br>Tubulin-tyrosine ligase inhibitor (ID 10602)
	432,2828	**KAIT**	ACE inhibitor (ID 8193, 7743)<br>Antioxidative (ID 8132)<br>DPP IV inhibitor (ID 3174)<br>DPP-III inhibitor (ID 9511)
	415,25606	**PSVI**	DPP IV inhibitor (ID 8862, 8895, 8920)
	459,28291	**ILDV**	ACE inhibitor (ID 9079)

		Neuropeptide (ID 10455)<br>DPP IV inhibitor (ID 8802)
481,26507	**VISY**	ACE inhibitor (ID 7684)<br>DPP IV inhibitor (ID 8897, 8920)
416,23124	**FLH**	ACE inhibitor (ID 11103, 9079, 11114)<br>Neuropeptide (ID 11101, 11104)<br>Antioxidative peptide (ID 3305)<br>DPP IV inhibitor (ID 8555, 8820)<br>DPP III inhibitor (ID 9502)<br>Pseudolysin inhibitor (ID 11213)
525,29128	**NVRH**	ACE inhibitor (ID 7628)<br>DPP IV inhibitor (ID 8594, 8851, 8883)
518,20333	**FLNC**	ACE inhibitor (ID 7832, 11103)<br>Neuropeptide (ID 11101)<br>DPP IV inhibitor (ID 8555, 8823)<br>DPP-III inhibitor (ID 9502)<br>Pseudolysin inhibitor (ID 11213)
547,336	**PFKR**	ACE inhibitor (ID 7586, 9211, 10718)<br>Antiviral peptide (ID 11018)<br>DPP IV inhibitor (ID 8814, 8854)<br>DPP-III inhibitor (ID 9505)<br>ACE2 inhibitor (ID 10460)<br>Hypouricemic peptide (ID 10709)
569,31751	**KRPF**	ACE inhibitor (ID 7582, 7586)<br>Immunomodulating (ID 3626)<br>Neuropeptide (ID 10116)<br>DPP IV inhibitor (ID 8518, 8814, 8854)<br>DPP-III inhibitor (ID 9505)<br>ACE2 inhibitor (ID 10460)<br>Neurolysin inhibitor (ID 10171,<br>Neprilysin inhibitor (ID10918)
515,1581	**TDEE**	Antithrombotic peptide (ID 3354)<br>Stimulating vasoactive substance release (ID 8329)<br>DPP IV inhibitor (ID 8898)<br>Glutamate carboxypeptidase inhibitor (ID 10622)<br>Glutamate carboxypeptidase II inhibitor (ID 10638, 10643)<br>Inhibitor of citrate lyase deacetylase (ID 10849)<br>Inhibitor of thymidylate synthase (ID 10850)
575,28518	**FFVY**	ACE inhibitor (ID 3492, 10027)<br>Antioxidative peptide (ID 8224)<br>Hypotensive peptide (ID 11112)<br>DPP IV inhibitor (ID 8929, 10028)<br>DPP-III inhibitor (ID 9509)<br>Inhibitor of tripeptidyl peptidase 2 (ID 10163)<br>Pseudolysin inhibitor (ID 10210)
430,24611	**PWK**	Antioxidative (ID 8190)<br>DPP IV inhibitor (ID 8676, 8865)
457,22822	**TITT**	ACE inhibitor (ID 7644)<br>DPP IV inhibitor (ID 8903, 8911)<br>Hypouricemic peptide (ID 10748)
439,23227	**WLV**	ACE inhibitor (ID 9107)

		Immunomodulating peptide (ID 11105)<br>Neuropeptide (ID 11102)<br>Antioxidative peptide (ID 11198)<br>DPP IV inhibitor (ID 8677, 8825)<br>PPARγ antagonist (ID 11205)
383,2151	**RAH**	ACE inhibitor (ID 7588, 7835)<br>DPP IV inhibitor (ID 8526, 8761)<br>Antioxidative (ID 7886)
423,23621	**HGLP**	ACE inhibitor (ID 7509, 7599, 7614, 10092)<br>DPP IV inhibitor (ID 3180, 8561)<br>Inhibitor of Lactocepin (ID 10620)
331,21861	**RR**	ACE inhibitor (ID 7741)<br>Antibacterial peptide (ID 10450)<br>Neuropeptide (ID 10454)<br>Hemolytic peptide (ID 10451)<br>Antiviral peptide (ID 10458)<br>DPP IV inhibitor (ID 8889)<br>DPP III inhibitor (ID 9485)<br>Leucyltransferase inhibitor (ID 10457)
552,33672	**AKRR**	ACE inhibitor (ID 7586, 7741, 10920)<br>Antibacterial peptide (ID 10450)<br>Neuropeptide (ID 10454)<br>Hemolytic peptide (ID 10451)<br>Antiviral peptide (ID 10458)<br>DPP IV inhibitor (ID 8814, 8889)<br>DPP III inhibitor (ID 9485)<br>Leucyltransferase inhibitor (ID 10457)<br>Neprilysin inhibitor (ID 10906)
625,32307	**MRRY**	ACE inhibitor (ID 3380, 7741)<br>Antibacterial peptide (ID 10450)<br>Neuropeptide (ID 10454)<br>Antioxidative peptide (ID 10051)<br>Hemolytic peptide (ID 10451)<br>Antiviral peptide (ID 10458)<br>DPP IV inhibitor (ID 8836, 8889)<br>DPP III inhibitor (ID 9479, 9485)<br>Leucyltransferase inhibitor (ID 10457)
375,20169	**WGL**	ACE inhibitor (ID 7599, 7613)<br>Antioxidative peptide (ID 9082)<br>DPP IV inhibitor (ID 8561, 8697)<br>Renin inhibitor (ID 10701)
425,21756	**VVW**	ACE inhibitor (ID 3486, 10924, 11400)<br>Antioxidative peptide (ID 8461)<br>DPP IV inhibitor (ID 3183, 8928)<br>Alpha-glucosidase inhibitor (ID 9387)<br>Inhibitor of tripeptidyl peptidase II (ID 19797)<br>Neprilysin inhibitor (ID 19714)<br>Xanthine oxidase inhibitor (ID 11193, 11397)
485,29299	**GPRR**	ACE inhibitor (ID 3537, 7512, 7741, 10903)<br>Antibacterial peptide (ID 10450)<br>Antithrombotic peptide (ID 3047, 3283)

			Neuropeptide (ID 10454)<br>Antioxidative peptide (ID 10927)<br>Hypotensive peptide (ID 10926)<br>Antiviral peptide (ID 10458)<br>DPP IV inhibitor (ID 3169, 8889, 10902)<br>DPP-III inhibitor (ID 9485, 9489)<br>Leucyltransferase inhibitor (ID 10457)<br>Neprilysin inhibitor (ID 10913)
	479,26568	**CKKT**	ACE inhibitor (ID 10921)<br>DPP IV inhibitor (ID 8813, 8816)<br>Neprilysin inhibitor (ID 10915)
	473,3211	**KAVR**	ACE inhibitor (ID 7628, 7743, 8951)<br>DPP IV inhibitor (ID 3174, 8594, 8764)<br>DPP-III inhibitor (ID 9511)
	637,30944	**FWRE**	ACE inhibitor (ID 10719)<br>DPP IV inhibitor (ID 8595, 8675)<br>PPARγ antagonist (ID 11207)<br>Neprilysin inhibitor (ID 10720)<br>Hypouricemic peptide (ID 10711)
	527,1607	**DDYD**	ACE inhibitor (ID 9072)<br>peptide regulating ion flow (ID 2749)<br>DPP IV inhibitor (ID 8973)<br>Glutamate carboxypeptidase II inhibitor ID10637
	288,20345	**LR**	ACE inhibitor (ID 9213)<br>Alpha-glucosidase inhibitor (ID 10360)<br>DPP-III inhibitor (ID 9478)<br>Renin inhibitor (ID 2842)<br>Hypouricemic peptide (ID 10712)<br>Neuroprotective peptide (ID 10959)
	229,15577	**PL**	ACE inhibitor (ID 7513)<br>DPP IV inhibitor (ID 8638)<br>Inhibitor of Xaa-Pro aminopeptidase (ID 10462)<br>Inhibitor of Lactocepin (ID 10463)
**BBPI-4**	490,2683	**PLNF**	ACE inhibitor (ID 7513, 7683, 7832, 8509)<br>DPP IV inhibitor (ID 8638, 8823, 8842)<br>Inhibitor of Xaa-Pro aminopeptidase (ID 10462)<br>Inhibitor of Lactocepin (ID 10463)
	490,26659	**LATW**	ACE inhibitor (IF 7585)<br>Antioxidant peptide (ID 8459)<br>DPP IV inhibitor (ID 3175, 8763, 8913)<br>DPP-III inhibitor (ID 9499)
	359,24053	**LAR**	ACE inhibitor (ID 7585, 7742)<br>DPP IV inhibitor (ID 3175)<br>DPP-III inhibitor (ID 9499)<br>Neprilysin inhibitor (ID 10907)
	322,18878	**FR**	ACE inhibitor (ID 7592)<br>DPP IV inhibitor (ID 8780)<br>DPP-III inhibitor (ID 9501)<br>Hypouricemic peptide (ID 10710)
	501,33856	**KIIQ**	DPP IV inhibitor (ID 8801, 8805, 8812)
	430,3029	**IKIG**	ACE inhibitor (ID 7595)

		DPP IV inhibitor (ID 8812)
456,29503	**IAPR**	ACE inhibitor (ID 3537, 7562, 7584, 7819)<br>DPP IV inhibitor (ID 3177, 8525)<br>DPP-III inhibitor (ID 9489)<br>Inhibitor of tripeptidyl peptidase II (ID 10750)<br>Inhibitor of peptidylprolyl isomerase (ID 10863)
431,25103	**ELIG**	ACE inhibitor (ID 7595)<br>Antioxidative peptide (ID 7888)<br>DPP IV inhibitor (ID 8821)
391,23896	**KMI**	DPP IV inhibitor (ID 8830)
468,2448	**KADL**	ACE inhibitor (ID 7743, 10408)<br>DPP IV inhibitor (ID 3174, 8757)<br>Alpha-glucosidase inhibitor (ID 9695)<br>DPP III inhibitor (ID 9511)
432,24737	**KADV**	ACE inhibitor (ID 7743)<br>DPP IV inhibitor (ID 3174, 8757)<br>Alpha-glucosidase inhibitor (ID 9695)<br>DPP III inhibitor (ID 9511)
532,20585	**DDPW**	Antioxidative (ID 8190)<br>DPP IV inhibitor (ID 8767, 8865)<br>Glutamate carboxypeptidase II inhibitor (ID 10637)
547,31063	**LRRC**	ACE inhibitor (ID 7741, 9213)<br>Antibacterial peptide (ID 10450)<br>Neuropeptide (ID 10454)<br>Hemolytic peptide (ID 10451)<br>Antiviral peptide (ID 10458)<br>DPP IV inhibitor (ID 8889)<br>Alpha-glucosidase inhibitor (ID 10360)<br>DPP III inhibitor (ID 9478, 9485)<br>Renin inhibitor (ID 2842)<br>Leucyltransferase inhibitor (ID 10457)<br>Hypouricemic peptide (ID 10712)<br>Neuroprotective peptide (ID 10959)
547,30803	**PRRP**	ACE inhibitor (ID 3537, 7582, 7741)<br>Antibacterial peptide (ID 10450)<br>Neuropeptide (ID 10116, 10454)<br>Hemolytic peptide (ID 10451)<br>Antiviral peptide (ID 10458)<br>DPP IV inhibitor (ID 8518, 8889)<br>DPP III inhibitor (ID 9485, 9489)<br>Leucyltransferase inhibitor (ID 10457)<br>Neurolysin inhibitor (ID 10171)<br>Neprilysin inhibitor (ID 10918)
380,18245	**VDF**	ACE inhibitor (ID 9074)<br>DPP IV inhibitor (ID 8915)<br>Glutamate carboxypeptidase II inhibitor (ID 10639)
465,28281	**IHPV**	ACE inhibitor (ID 7842)<br>DPP IV inhibitor (ID 8520, 8800)<br>DPP III inhibitor (ID 9496, 9497)
416,15674	**GSKC**	ACE inhibitor (ID 7607)<br>DPP IV inhibitor (ID 8894)

598,33399	**IFRY**	ACE inhibitor (ID 3380, 7592, 7593, 11012)<br>Antioxidative peptide (ID 10051)<br>Hypotensive peptide (ID 11013)<br>DPP IV inhibitor (ID 8780)<br>DPP-III inhibitor (ID 9501)<br>Hypouricemic peptide (ID 10710)
438,1403	**EMH**	DPP IV inhibitor (ID 8829)
265,15521	**VF**	ACE inhibitor (ID 3384)<br>DPP IV inhibitor (ID 8917)<br>Calpain 1 inhibitor (ID9918)<br>Inhibitor of tripeptidyl peptidase II (ID 9920)<br>Inhibitor of neprilysin-2 (ID 10095)
366,20285	**ISF**	ACE inhibitor (ID 7685)<br>DPP IV inhibitor (ID 8891)<br>Renin inhibitor (ID 9432)
395,22798	**FTK**	DPP IV inhibitor (ID 8904)<br>Renin inhibitor (ID 2835)
378,20416	**PVY**	ACE inhibitor (ID 3492)<br>antioxidative peptide (ID 8224)<br>Hypotensive peptide (ID 11112)<br>DPP IV inhibitor (ID 8864, 8929)<br>DPP-III inhibitor (ID 9509)<br>Inhibitor of tripeptidyl peptidase 2 (ID 10163)
346,23487	**LLT**	Antioxidative peptide (ID 10749)<br>DPP IV inhibitor (ID 3182, 8824)<br>Inhibitor of Lactocepin (ID 10619)<br>Inhibitor of cytosol alanyl aminopeptidase (ID 10615)<br>Hypouricemic peptide (ID 10741)
559,35831	**KIIW**	ACE inhibitor (ID 7544)<br>Hypotensive peptide (ID 11192)<br>DPP IV inhibitor (ID 8801, 8807, 8812)
401,28874	**IRI**	ACE inhibitor (ID 3258)<br>Antioxidative peptide (ID 8215)<br>DPP IV inhibitor (ID 8806, 8884)<br>CaMPDE inhibitor (ID 8247)<br>Renin inhibitor (ID 8246)
322,1754	**VFG**	ACE inhibitor (ID 3384, 7605)<br>DPP IV inhibitor (ID 8917)<br>Calpain 1 inhibitor (ID9918)<br>Inhibitor of tripeptidyl peptidase II (ID 9920)<br>Inhibitor of neprilysin-2 (ID 10095)<br>Neprilysin inhibitor (ID 10546)
472,35111	**KIIV**	DPP IV inhibitor (ID 8801, 8812)
432,28312	**KAIT**	ACE inhibitor (ID 8193, 7743)<br>Antioxidative (ID 8132)<br>DPP IV inhibitor (ID 3174)<br>DPP-III inhibitor (ID 9511)
493,13766	**DAYC**	ACE inhibitor (ID 3563, 7606)<br>Antioxidative (ID 7866)<br>DPP IV inhibitor (ID 8765)<br>DPP-III inhibitor (ID 9492)

		Tubulin-tyrosine ligase inhibitor (ID 10604)<br>Glutamate carboxypeptidase II inhibitor (ID 10636)
415,25615	**PSVI**	DPP IV inhibitor (ID 8862, 8895, 8920)
459,28301	**ILDV**	ACE inhibitor (ID 9079)<br>Neuropeptide (ID 10455)<br>DPP IV inhibitor (ID 8802)
481,26545	**VISY**	ACE inhibitor (ID 7684)<br>DPP IV inhibitor (ID 8897, 8920)
547,29528	**RRSE**	ACE inhibitor (ID 7741)<br>Antibacterial peptide (ID 10450)<br>Neuropeptide (ID 10454)<br>Hemolytic peptide (ID 10451)<br>Antiviral peptide (ID 10458)<br>DPP IV inhibitor (ID 8889)<br>DPP III inhibitor (ID 9485)<br>Leucyltransferase inhibitor (ID 10457)
344,25564	**VIL**	ACE inhibitor (ID 9070)<br>Neuropeptide (ID 10455)<br>DPP IV inhibitor (ID 8802, 8920)
596,33247	**RRSR**	ACE inhibitor (ID 7741)<br>Antibacterial peptide (ID 10450)<br>Neuropeptide (ID 10454)<br>Hemolytic peptide (ID 10451)<br>Antiviral peptide (ID 10458)<br>DPP IV inhibitor (ID 8889)<br>DPP III inhibitor (ID 9485)<br>Leucyltransferase inhibitor (ID 10457)
547,33545	**KRPF**	ACE inhibitor (ID 7582, 7586)<br>Immunomodulating (ID 3626)<br>Neuropeptide (ID 10116)<br>DPP IV inhibitor (ID 8518, 8814, 8854)<br>DPP-III inhibitor (ID 9505)<br>ACE2 inhibitor (ID 10460)<br>Neurolysin inhibitor (ID 10171,<br>Neprilysin inhibitor (ID 10918)
515,15883	**TDEE**	Antithrombotic peptide (ID 3354)<br>Stimulating vasoactive substance release (ID 8329)<br>DPP IV inhibitor (ID 8898)<br>Glutamate carboxypeptidase inhibitor (ID 10622)<br>Glutamate carboxypeptidase II inhibitor (ID 10638, 10643)<br>Inhibitor of citrate lyase deacetylase (ID 10849)<br>Inhibitor of thymidylate synthase (ID 10850)
580,29843	**HVRF**	ACE inhibitor (ID 3489, 7628, 10692)<br>DPP IV inhibitor (ID 8594, 8797)<br>DPP III inhibitor (ID 9490)<br>Leucyltransferase inhibitor (ID 10547)<br>ACE 2 activator (ID 10684)
279,17157	**FL**	ACE inhibitor (ID 11103)<br>Neuropeptide (ID 11101)<br>DPP IV inhibitor (ID 8555)<br>DPP-III inhibitor (ID 9502)

		Pseudolysin inhibitor (ID 11213)
597,26887	**FYFV**	Alpha-amylase inhibitor (ID 10728)<br>ACE inhibitor (ID 3556)<br>antioxidative peptide (ID 10471, 10732)<br>Hypotensive peptide (ID 10731)<br>DPP IV inhibitor (ID 8935)<br>DPP-III inhibitor (ID 9480)<br>Alpha-glucosidase inhibitor (ID 10730)
575,28664	**FFVY**	ACE inhibitor (ID 3492, 10027)<br>antioxidative peptide (ID 8224)<br>Hypotensive peptide (ID 11112)<br>DPP IV inhibitor (ID 8929, 10028)<br>DPP-III inhibitor (ID 9509)<br>Inhibitor of tripeptidyl peptidase 2 (ID 10163)<br>Pseudolysin inhibitor (ID 10210)
575,28692	**YVFF**	ACE inhibitor (ID 3384, 9077, 10027)<br>DPP IV inhibitor (ID 8917, 8946, 10028)<br>Calpain 1 inhibitor (ID 9918)<br>Inhibitor of tripeptidyl peptidase II (ID 9920)<br>Inhibitor of neprilysin-2 (ID 10095)<br>Pseudolysin inhibitor (ID 10210)
442,24	**HAKS**	ACE inhibitor (ID 10920)<br>DPP IV inhibitor (ID 3184, 8815)<br>Neprilysin inhibitor (ID 10906)<br>Alanine carboxypeptidase inhibitor (ID 10808)
358,27156	**ILL**	ACE inhibitor (ID 9079)<br>Neuropeptide (ID 10455)<br>DPP IV inhibitor (ID 3182, 8802)<br>Inhibitor of Lactocepin (ID 10619)<br>Inhibitor of cytosol alanyl aminopeptidase (ID 10615)
481,26683	**SVYI**	ACE inhibitor (ID 3492, 9038, 11114)<br>Neuropeptide (ID 11115)<br>Antioxidative peptide (ID 8224)<br>Hypotensive peptide (ID 11112)<br>DPP IV inhibitor (ID 8895, 8929, 8938)<br>DPP-III inhibitor (ID 9509, 9510)<br>Inhibitor of tripeptidyl peptidase 2 (ID 10163)
477,30677	**VFIV**	ACE inhibitor (ID 3384)<br>DPP IV inhibitor (ID 8917)<br>Calpain 1 inhibitor (ID9918)<br>Inhibitor of tripeptidyl peptidase II (ID 9920)<br>Inhibitor of neprilysin-2 (ID 10095)
392,25567	**LLF**	ACE inhibitor (ID 3551, 7807)<br>Antioxidative peptide (ID 10877)<br>Antihypertensive peptide (ID 10876)<br>DPP IV inhibitor (ID 3182)<br>Inhibitor of Lactocepin (ID 10619)<br>Pseudolysin inhibitor (ID 8313)<br>Inhibitor of cytosol alanyl aminopeptidase (ID 10615)
331,21892	**RR**	ACE inhibitor (ID 7741)<br>Antibacterial peptide (ID 10450)

		Neuropeptide (ID 10454)<br>Hemolytic peptide (ID 10451)<br>Antiviral peptide (ID 10458)<br>DPP IV inhibitor (ID 8889)<br>DPP III inhibitor (ID 9485)<br>Leucyltransferase inhibitor (ID 10457)
375,20181	**WGL**	ACE inhibitor (ID 7599, 7613)<br>Antioxidative peptide (ID 9082)<br>DPP IV inhibitor (ID 8561, 8697)<br>Renin inhibitor (ID 10701)
425,21772	**VVW**	ACE inhibitor (ID 3486, 10924, 11400)<br>Antioxidative peptide (ID 8461)<br>DPP IV inhibitor (ID 3183, 8928)<br>Alpha-glucosidase inhibitor (ID 9387)<br>Inhibitor of tripeptidyl peptidase II (ID 19797)<br>Neprilysin inhibitor (ID 19714)<br>Xanthine oxidase inhibitor (ID 11193, 11397)
485,29321	**GPRR**	ACE inhibitor (ID 3537, 7512, 7741, 10903)<br>Antibacterial peptide (ID 10450)<br>Antithrombotic peptide (ID 3047, 3283)<br>Neuropeptide (ID 10454)<br>Antioxidative peptide (ID 10927)<br>Hypotensive peptide (ID 10926)<br>Antiviral peptide (ID 10458)<br>DPP IV inhibitor (ID 3169, 8889, 10902)<br>DPP-III inhibitor (ID 9485, 9489)<br>Leucyltransferase inhibitor (ID 10457)<br>Neprilysin inhibitor (ID 10913)
479,26571	**CKKT**	ACE inhibitor (ID 10921)<br>DPP IV inhibitor (ID 8813, 8816)<br>Neprilysin inhibitor (ID 10915)
473,32099	**KAVR**	ACE inhibitor (ID 7628, 7743, 8951)<br>DPP IV inhibitor (ID 3174, 8594, 8764)<br>DPP-III inhibitor (ID 9511)
